# Sensor-Model-Based Trajectory Optimization for UAVs to Enhance Detection Performance: An Optimal Control Approach and Experimental Results

**DOI:** 10.3390/s23020664

**Published:** 2023-01-06

**Authors:** Markus Zwick, Matthias Gerdts, Peter Stütz

**Affiliations:** 1 Institute of Flight Systems, Universität der Bundeswehr München, 85579 Neubiberg, Germany; 2 Institute of Applied Mathematics and Scientific Computing, Universität der Bundeswehr München, 85579 Neubiberg, Germany

**Keywords:** aerial reconnaissance, trajectory optimization, optimal control, sensor performance model

## Abstract

UAVs are widely used for aerial reconnaissance with imaging sensors. For this, a high detection performance (accuracy of object detection) is desired in order to increase mission success. However, different environmental conditions (negatively) affect sensory data acquisition and automated object detection. For this reason, we present an innovative concept that maps the influence of selected environmental conditions on detection performance utilizing sensor performance models. These models are used in sensor-model-based trajectory optimization to generate optimized reference flight trajectories with aligned sensor control for a fixed-wing UAV in order to increase detection performance. These reference trajectories are calculated using nonlinear model predictive control as well as dynamic programming, both in combination with a newly developed sensor performance model, which is described in this work. To the best of our knowledge, this is the first sensor performance model to be used in unmanned aerial reconnaissance that maps the detection performance for a perception chain with a deep learning-based object detector with respect to selected environmental states. The reference trajectory determines the spatial and temporal positioning of the UAV and its imaging sensor with respect to the reconnaissance object on the ground. The trajectory optimization aims to influence sensor data acquisition by adjusting the sensor position, as part of the environmental states, in such a way that the subsequent automated object detection yields enhanced detection performance. Different constraints derived from perceptual, platform-specific, environmental, and mission-relevant requirements are incorporated into the optimization process. We evaluate the capabilities of the sensor performance model and our approach to sensor-model-based trajectory optimization by a series of simulated aerial reconnaissance tasks for ground vehicle detection. Compared to a variety of benchmark trajectories, our approach achieves an increase in detection performance of 4.48% on average for trajectory optimization with nonlinear model predictive control. With dynamic programming, we achieve even higher performance values that are equal to or close to the theoretical maximum detection performance values.

## 1. Introduction

Unmanned aerial vehicles (UAVs) with imaging sensors in the visual or infrared spectrum are increasingly used in various fields in civil, commercial and military applications. Examples include surveillance and reconnaissance missions [1,2], environmental monitoring [3,4], aerial photogrammetric mapping [5,6], or search and rescue missions [7,8,9,10]. In all these applications, a high detection performance (a measure to describe the accuracy of localization and classification of objects within the sensor footprint) is aspired to perform the mission successfully. A high detection performance imposes a high demand on the capabilities of the sensor data processing and analysis algorithms, especially if the sensor data are analyzed in an automated manner directly on board the UAV. Changing environmental conditions (e.g., brightness, visibility conditions) as well as variable operational and parameter settings can have a negative impact on sensor data acquisition and the subsequent processing chain, which can ultimately lead to a degradation of the detection performance [11]. Moreover, it is important to quantitatively determine the confidence in the measurement results. This is particularly relevant if only the processed results of the automated object detection are transmitted to a human (e.g., UAV operator), who has to deduce further action from these results [12].

In addition to automated sensory data acquisition and object detection, we also address the optimization of UAV flight trajectories in the following. In this work, we reuse the two optimization methods *nonlinear model predictive control* (NMPC) and *dynamic programming & optimal control* (DP&OC), which were proposed in our previous works [13,14]. NMPC is a well-established method for UAV trajectory optimization and is used for example by [15,16,17,18]. For the application of DP&OC for path and trajectory planning, we refer to [19,20,21,22]. There is extensive work in the literature on algorithms for general trajectory optimization. For a comprehensive overview of this topic, we refer the reader to [23,24].

**Figure 1:**
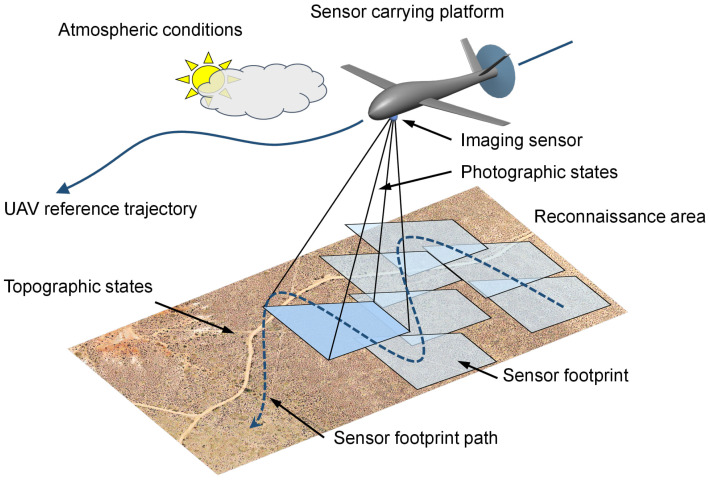
Illustration of relevant influencing factors on sensor-model-based trajectory optimization. Adapted from [13].

Figure 1 is intended to give an overview of perceptual, platform-specific, environmental, and mission-related aspects and influencing factors that have to be considered for trajectory optimization. These aspects will be discussed in the respective sections of this paper.

### 1.1. State of the Art

Various publications can be found in the literature examining the detection performance as a function of environmental conditions. Examples are given in the following:

The authors in [25] describe the influences of different environmental states including topographic, atmospheric, and photographic conditions on the detection performance of various perception chains for aerial surveillance and reconnaissance. The effect of the environmental states on the detection performance was mapped by sensor performance models. The goal is to dynamically find and select the best-performing perception chain by the performance models depending on the prevailing environmental conditions.

In [26], the authors investigate the impact of the ground sample distance (GSD) on the detection performance of three different deep learning-based object detectors applied to aerial reconnaissance. The detectors used include YOLOv2 and Faster R-CNN. It was determined that the GSD affects the achieved average precision (AP) and a deviation from a specific GSD value resulted in a deterioration of the AP.

In [27], an approach is presented to automatically detect injured humans in images taken by a UAV. The authors could substantially improve the detection performance of different object detectors due to the consideration of photographic states with respect to height and pitch.

### 1.2. Research Gap

The photographic states, comprising the *elevation angle* (angle between the horizontal plane and the line of sight of the sensor), as well as the *ground sample distance* affect the detection performance of perception chains used in aerial reconnaissance [25,28]. Here, the perception chain [29] consists of an imaging sensor, as well as downstream algorithms for data processing and automated object detection.

The following examples of aerial reconnaissance either lack a sensor performance model or use only a highly simplified model. This makes detailed and realistic coordination of UAV and sensor planning, as well as the calculation of the expected detection performance for a real perception chain, impossible.

In [30], the authors present an approach to a UAV-based search for human victims with imaging sensors. The UAV trajectory optimization is performed by model predictive control. A person is considered to be detected if he or she is covered by the field of view of the camera that is mounted to the UAV. It is found that the trajectory optimization lacks a detailed sensor performance model that takes into account the detection performance of the deployed perception chain. According to [27], the elevation angle has an impact on the detection performance and therefore should be considered in more detailed trajectory planning.

The authors in [31] propose a concept to plan optimized UAV trajectories to recognize objects on the ground. To execute the aerial reconnaissance task, the UAV is equipped with an electro-optical/infrared sensor system. The detection performance of the perception chain is modeled utilizing the “National Imagery Interpretability Rating Scale” (NIIRS), which leads to a major simplification of its capabilities and limitations. The detection performance is essentially determined only by the ground sample distance. Furthermore, atmospheric and topographic conditions in the reconnaissance area are also neglected, and consequently their influence on detection performance.

### 1.3. Research Problem

The sensor performance model maps selected environmental states to the detection performance of a specific perception chain. These environmental states include topographic, atmospheric, and photographic states (e.g., elevation angle and ground sample distance) [25].

From the research gap and to our knowledge: the selective manipulation of the photographic states by a coordinated UAV and sensor movement in order to enhance the detection performance determined by a sensor performance model has not yet been sufficiently investigated for the field of aerial reconnaissance.

To address this research problem, we have developed an innovative concept that we present in the following.

### 1.4. Innovative Contribution and Novelty in This Work

Our innovative contribution to the research problem is the development of a functional concept for *sensor-model-based trajectory optimization* in the field of unmanned aerial reconnaissance, which we presented for the first time in our previous works [13,14].

To our knowledge, this is the first concept that utilizes detailed sensor performance models of different perception chains in order to generate optimal UAV trajectories to increase detection performance. The concept is based on a sensor performance model that maps the dependence of the detection performance for various environmental conditions. By employing different optimization methods, optimal UAV reference (setpoint) trajectories are generated from this model under consideration of multiple constraints (e.g., flight dynamic limitations of the UAV). The optimization specifically exploits the dependencies of the photographic states *elevation angle* and *ground sample distance* (as part of the environmental states) on the detection performance to generate a UAV reference flight trajectory along with a coordinated sensor footprint movement on the ground. In our previous works [13,14], we were able to enhance detection performance resulting from the optimized UAV reference trajectories compared to those of benchmark trajectories. Thus, the validity and capability of our concept could be proven.

The novelty in this work is the development and evaluation of a new sensor performance model for a perception chain with a deep learning-based object detector. We evaluate the performance model in different simulated experiments by generating optimal UAV reference trajectories, using two different optimization methods. The resulting detection performances from the optimized reference trajectories are compared with the detection performances that would arise from various benchmark trajectories.

An additional novelty is to model the state transitions of the discrete optimization method *dynamic programming & optimal control* by Dubins paths in order to achieve more realistic UAV dynamics than obtained in our previous work [14]. With this, it can be guaranteed that UAV reference trajectories are generated that meet predefined roll angle limitations.

### 1.5. Outline

This paper is structured as follows: in Section 2 we briefly describe the use of *coverage path planning* for sensor control. This is followed by the introduction of a newly developed *sensor performance model* to map the detection performance of a perception chain comprising a deep learning object detector. We proceed with the explanation of *perception maps* in Section 2.2.3 and briefly explain the basics of *optimal control* in Section 2.3. This is the basis for trajectory optimization with *nonlinear model predictive control* in Section 2.4.2 and *dynamic programming and optimal control* in Section 2.5.2. We validate our approach in Section 3 and summarize the results in Section 4 and Section 5.

## 2. Materials and Methods

In our approach, the spatio-temporal positioning of the sensor footprint on the ground is separated from the computation of the optimized flight trajectory and performed sequentially. For this, the path of the sensor footprint on the ground is determined using coverage path planning, and then the UAV’s flight trajectory is optimized with respect to this footprint path. The separation is considered necessary to reduce the high complexity that a fully combined planning and optimization of the sensor control and the flight trajectory would entail.

A basic problem of planning theory in optimal approaches is that many general planning problems belong to the class of NP-hard problems [32], which means that there are no known polynomial-time algorithms for solving this class of problems. An alternative is to restrict to suboptimal solutions using heuristic techniques [33]. For this reason, in our approach, the sensor footprint positioning is planned first (Section 2.1) and the movement of the UAV is adapted and optimized (Section 2.3) accordingly, while complying with numerous constraints related to mission, sensor platform, environmental and perceptual aspects.

The goal is to generate reference trajectories for a UAV that are aligned with the spatio-temporal sensor footprint positioning. The reference trajectory defines the setpoints for the autopilot on board the UAV to perform the actual reconnaissance flight guidance, which is not covered in this work. The spatio-temporal progression of the reference trajectory has to take flight dynamic limitations of the UAV into account in order to model realistic flight behavior. This includes roll angle and roll rate limitations, as well as a constant airspeed for the fixed-wing UAV. For the reference trajectory, continuous curvature ($C^{2}$-continuous function) is required.

### 2.1. Coverage Path Planning for Sensor Control

*Coverage path planning* (CPP) is often the first step in processing a reconnaissance task. The purpose of CPP is to define the spatial and temporal positioning of the sensor footprint in the area to be reconnoitered (see *area reconnaissance scenario* in Section 3.3) or along a predefined route (see *route reconnaissance scenario* in Section 3.2). The predefined area or route is deterministically and completely reconnoitered by utilizing CPP, with the goal to detect objects of interest with a high detection performance. Since it is assumed that no prior information is available about the quantity and location of objects in the area, a systematic search approach using CPP is utilized. In this work, ground vehicles are the objects of interest and the focus is on their detection.

With CPP, a cellular discretization of the reconnaissance area is made according to the boustrophedon decomposition [34], combined with a back-and-forth planning of the sensor footprint as described in [35]. The sweep width $w_{fp}$ (1) for the CPP is calculated from the width of the sensor footprint on the ground. It is composed of the target ground sample distance (GSD) $gsd_{ref}$ and the resolution of the imaging sensor $R_{sens}$. The shape of the target sensor footprint on the ground is simplified as a square with edge length $w_{fp}$.

(1) $$w_{fp}=gsd_{ref}\cdot R_{sens}$$

The euclidean distance $d_{fp}$ (2) between the centers of two successive sensor footprints is determined by the setpoint of the sensor footprint velocity $v_{fp}$ and the time step interval Δt.

(2) $$d_{fp}=v_{fp}\cdot \Delta t$$

**Figure 2:**
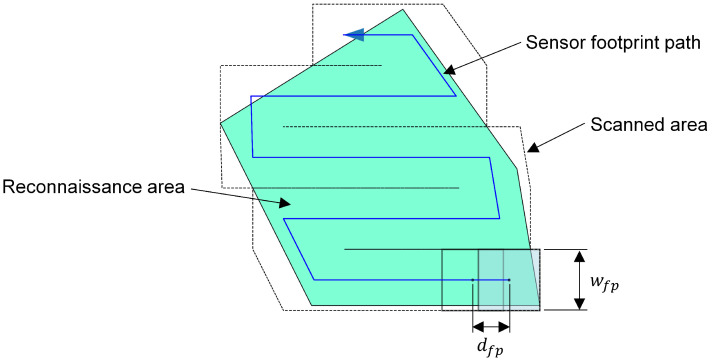
Principle of coverage path planning for a reconnaissance area (green). The sensor footprint path defines the positioning of the individual sensor footprints (pale blue). The size of the footprint is defined by $w_{fp}$ and the Euclidean distance between footprints is determined by $d_{fp}$. The black dotted line marks the scanned area.

Figure 2 shows an example of coverage path planning applied to a reconnaissance area (green). The result is the sensor footprint path (blue), which defines the position of the individual sensor footprints. Furthermore, the first sensor footprint (pale blue), as well as an overlapping second footprint (black outline) are displayed.

Table 1 lists the parameter settings that are relevant for coverage path planning in this work. Parameters marked “predefined” were determined based on previous work or studies, which will not be discussed here.

By applying CPP, we obtain a sequence of concatenated sensor footprints. Moreover, the procedure determines the number, position and order of the footprints, which becomes important for the generation of the *perception maps* in Section 2.2.3.

### 2.2. Sensor Performance Models

**Figure 3:**
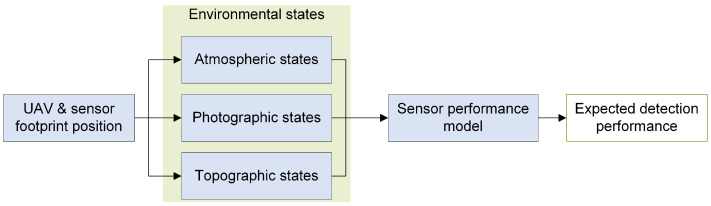
The sensor performance model maps selected environmental states to the expected detection performance of a specific perception chain (not displayed). These environmental states comprise atmospheric, photographic, and topographic conditions resulting from the positioning of the UAV and the sensor footprint on the ground.

In this section, the concept and design of the applied *sensor performance models* will be discussed. A sensor performance model [25], as depicted in Figure 3 and used in this work, maps the influence of certain *environmental states* on the *expected detection performance* with respect to a specific *perception chain* [29]. The perception chain comprises the essential hardware and software components from data acquisition to data evaluation. It incorporates an electro-optical or infrared sensor for data acquisition, components for sensor data processing, and algorithms for automated object detection comprising localization and classification. The environmental states depend on the UAV and/or sensor footprint position, which will be discussed in Section 2.2.2. The *topographic states* refer to the content of the sensor footprint. The *atmospheric states* take into account, among other things, the local weather conditions in the UAV’s operational area, and the *photographic states* depend on the position of the UAV relative to the sensor footprint on the ground.

Sensor performance models enable a quantitative prediction of the detection performance under the influence of selected environmental conditions. The value of the predicted detection performance $p_{det}$ ranges from 0 to 1. A high value corresponds to a good algorithm performance of the used perception chain, while a low value implies poor performance. Thus, it is a quantitative representation of the trustworthiness in the measurement result of a detection affected by the environment states.

In the following, two different performance models for vehicle detection are presented. In Section 2.2.1, a newly developed model is explained that represents a perception chain comprising a deep learning-based object detector. In contrast, Section 2.2.2 discusses a performance model relying on a machine learning-based object classifier. This performance model has already been introduced in our previous work [14]. Still, it is briefly explained again in this work as it is applied in Section 3 for the validation of the partially new developed trajectory optimization with *dynamic programming* (Section 2.5).

#### 2.2.1. Deep Learning Based Object Detector

The objective of this section is to develop a *sensor performance model* to map the detection performance of a perception chain utilizing YOLOv3, a deep learning-based object detector. The performance model builds on a model dataset for vehicle detection, as well as a trained YOLOv3 detector from the work of [36]. Although there are more recent YOLO versions nowadays, we develop the sensor performance model for the YOLOv3 detector. The reason is that we build on the dataset of [36], for which object detection has already been performed with this detector. However, the concept for this performance model is not limited to that specific detector version, which will be briefly explained at the end of this section.

There are several reasons, which are summarized in the following, for choosing YOLOv3 as an object detector to be utilized in a perception chain, and countless applications can be found in the literature.

- Although now superseded by newer versions, YOLOv3 is still an efficient and high-performing object detector [37,38].
- The free code base of the YOLOv3 detector and the availability of public and annotated datasets (e.g., the UAVDT dataset [39]) have contributed to the widespread use of this detector.
- YOLOv3 enables real-time image-based object detection on commercially available hardware [40], which is especially advantageous for use on board the UAV.

In the past, Krump et al. [41] trained a YOLOv3 object detector for ground-based vehicle detection using the images from the UAVDT dataset [39]. This dataset features a large number of labeled aerial images and a high variation with respect to flight altitude, viewing angle, and environmental conditions (e.g., daylight, night, fog).

In [36], Krump & Stütz describe the generation of a custom image dataset for vehicle detection consisting of approximately 3300 images taken by a UAV with an electro-optical sensor. In addition to the atmospheric conditions, the vertical and horizontal distance between the UAV and the vehicles, as well as the bounding box of the vehicles were recorded and annotated. For this dataset, which will be referred to as the *K&S dataset* in the following, the aerial images were taken by varying the altitude (from 15 m to 90 m) and horizontal distance (from 0 m to 80 m) between the UAV and the vehicles. In order to achieve a wide variation of atmospheric conditions (see Table 2), the images were taken at different times of day and weather conditions. Furthermore, multiple vehicles were captured on different road surfaces and against varying backgrounds. In contrast to the UAVDT dataset, for the K&S dataset, the altitude above ground and the horizontal distance were measured and annotated, from which the ground sample distance and the elevation angle can be calculated.

**Figure 4:**
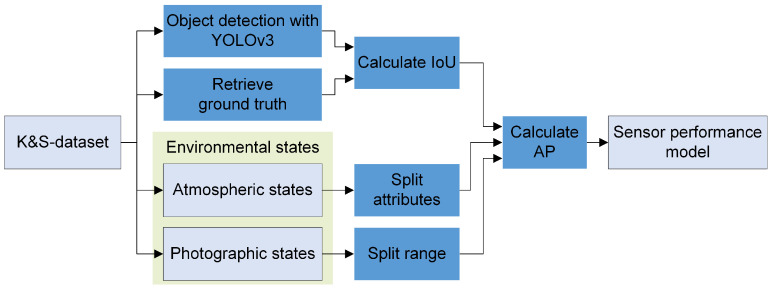
Procedure for generating the sensor performance model utilizing a YOLOv3 object detector. States, the dataset, and the performance model are shown in light gray, actions are colored in blue. The green rectangle marks the environmental states, consisting of the atmospheric and photographic states.

The trained YOLOv3 object detector from [41] was applied to the annotated K&S dataset by Krump & Stütz in [36]. The detector performed object localization and classification for each image (depicted in Figure 4) and the results were recorded.

To transfer these results in a sensor performance model for this work, we define the GSD and the elevation angle as the independent variables or inputs of the performance model, whereas the expected detection performance is the dependent variable or output of the model. The GSD and the elevation angle are both parts of the photographic state and suitable for targeted affecting of the detection performance by selectively adjusting the sensor/UAV position, as well as the sensor’s field of view [42]. We conducted the following steps to develop the performance model, which is also depicted graphically in Figure 4:

In the first step, we calculated the *intersection over union* (IoU) from the bounding box of the labeled ground truth and the predicted bounding box from the recorded localization of the YOLOv3 object detector. The IoU is a measure that scores the overlap between two bounding boxes. We set a threshold of 0.5 for the IoU and determined whether a detection is true positive (TP), false positive (FP), or false negative (FN). The IoU is a common metric in the field of object detection to evaluate the accuracy of localization.

Next, from the K&S dataset, we calculated the elevation angle and GSD (both part of the photographic states) for each individual image utilizing the annotated data regarding the altitude above ground and the horizontal distance between the vehicle and the UAV. We then divided the dataset into 16 individual datasets by splitting the range of the GSD and the elevation angle into four intervals each. The interval sizes were chosen such that each region could be assigned approximately the same number of images.

Further, we divided the K&S dataset into sub-datasets with different compositions of the complementary atmospheric states (see Table 2). It is mentioned that the sensor performance model with the composition {*autumn*, *day*, *clear*, *wet*, *covered*} is used in this work.

Thus far, for each image in the K&S dataset, the IoU has been determined. Then, based on their annotated data, the images were divided into sub-datasets by splitting the photographic states. This was carried out likewise for all complementary atmospheric states.

In the last step, from TP, FP, and FN, we computed *precision* and *recall* for each sub-dataset and calculated the *precision-recall curve*. From this, we obtained die *average precision* (AP) as the area under the precision-recall curve. The AP has a high value if both precision and recall are high and a low value if either of them is low, with its value ranging between 0 and 1. For the sensor performance model with a deep learning object detector, we define AP as the measure of the detection performance. Therefore, a high AP corresponds to a high detection performance and vice versa.

**Figure 5:**
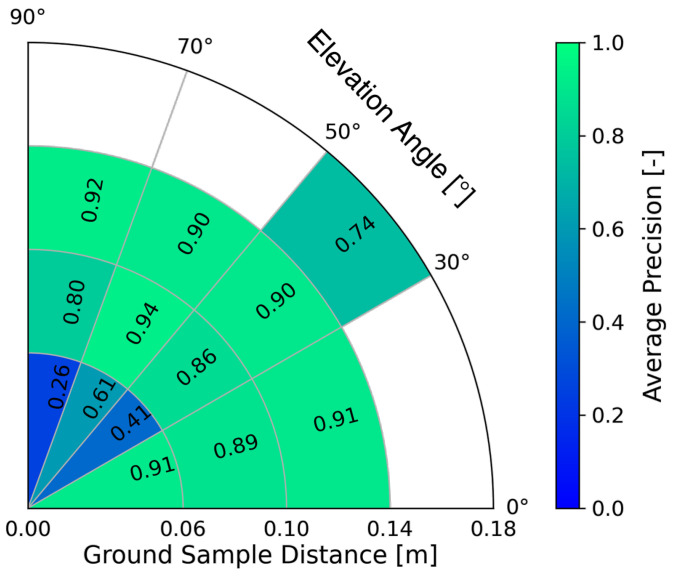
Illustration of the average precision (color-coded) for different interval ranges of the ground sample distance and the elevation angle corresponding to a specific composition of the environmental state.

Figure 5 shows the result of the AP for a specific configuration of the environmental states. This also shows the interval ranges for the GSD and elevation angle, resulting in 16 sections. White fields indicate that there is no image data available for this case. It is also evident from the plot that the AP varies depending on the GSD and the elevation angle. We observed similar behavior with different compositions of the atmospheric conditions. Therefore, by employing a newer YOLO detector (e.g., YOLOv4 [43]), we also expect the AP to be dependent on the GSD and the elevation angle, which means that our approach to modeling the sensor performance model will still hold. However, when applying a newer YOLO version, the AP values in the individual intervals are expected to shift towards higher values, as shown by an investigation of [44] for YOLOv3, YOLOv4 and YOLO5l.

#### 2.2.2. Machine Learning Based Object Classifier

**Figure 6:**
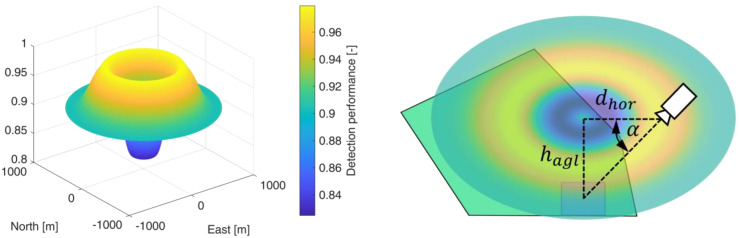
Representation of a perception map from the CC sensor performance model as a 3-dimensional plot (**left**) and the same map in a planar representation (**right**), with reference to the corresponding sensor footprint (pale blue square) on the ground. The elevation angle α is determined by the horizontal distance $d_{hor}$ and the altitude above ground $h_{agl}$. The color-coding of the perception map corresponds to the predicted detection performance. Light colors represent high performance values, while darker colors correlate with lower values.

The development of the sensor performance model with a machine learning-based object classifier is described in [25]. For this, synthetic model datasets comprising visual and infrared images of vehicles in different environments were generated in a simulation environment. These datasets also include the associated environment state vector, which holds the ground truth of the simulated *atmospheric*, *photographic*, and *topographic* conditions at the moment of image data acquisition. The environment state vector consists of the following states, as stated in Section 2.2:

- Atmospheric states: cloudiness, fog, precipitation and lightening conditions defined by the time of day and month.
- Topographic states: land cover (roads, meadow, water, vegetation and buildings) and the surface roughness within the sensor footprint.
- Photographic states: ground sample distance and the sensor elevation angle (see Figure 6 right plot).

Each of these states affects the acquired sensor data and the subsequently used computer vision algorithms and thus influences the detection performance. In [25], various algorithms with machine learning-based object classifiers for vehicle detection were used to evaluate the sensor data, in particular *classification cascade* (CC), *deformable part model* (DPM), *template matching* (TM), and *binary large object* (BLOB). The detection performance results from the data evaluation and is expressed as the F1-score, which includes both *precision* and *recall* of the algorithm as a statistical quantity. In a final step, neural networks were trained to predict the *expected detection performance* based on the environment state vector. For each perception chain with its object classifier CC, TM, DPM or BLOB, an individual sensor performance model was created. Further information on development, implementation and validation can be found in more detail in [25,45].

Among the sensor performance models presented in this section, only the model based on *classification cascade* (CC) is considered further in this work.

#### 2.2.3. Perception Maps

In previous work [42], we have shown that specific environmental states exist which can be manipulated in a targeted manner in order to deliberately influence and, in the best case, enhance the detection performance. In this way, the negative effects on the detection performance by uncontrollable conditions, such as visibility, daylight, or precipitation, can be compensated or mitigated. The set of relevant mutable states includes the *elevation angle* and the *ground sample distance*. Both states can be selectively adjusted within limits by changing the sensor/UAV position relative to the sensor footprint location on the ground and by adapting the sensor’s field of view. This is the basis for sensor-model-based trajectory optimization. Here, the elevation angle and the field of view are specifically altered in order to increase the detection performance while considering numerous constraints related to the mission, sensor platform, and perceptual aspects.

The sensor performance model used implicitly maps the detection performance by a neural network (Section 2.2.2) or as a section-wise defined function (Section 2.2.1). Therefore, the mapping is not explicitly available as a multivariate function that permits a direct evaluation of the respective environment states on the detection performance. For this reason, we use the concept of the *perception map* (PM) that was developed in [14] and is briefly explained in the following.

A PM represents the course of the detection performance (shaped as a potential field) in a 2-dimensional plane, which coincides with the plane of motion of the UAV in a fixed altitude above ground $h_{agl}$. For each individual sensor footprint, defined by coverage path planning in Section 2.1, an individual PM is created. Each PM is rotation symmetric and circular with diameter $d_{pm}$ (see Table 3) and with its center perpendicular above the center of the corresponding sensor footprint. The PM is obtained by selectively varying the sensor/UAV position in the 2-dimensional plane, which leads to a change in the elevation angle. The position of the sensor footprint permits the determination of the topographic state within the footprint using a geographic information system (GIS). Together with the atmospheric conditions prevailing in the PM, the detection performance can be calculated for the specific elevation angle using the sensor performance model. The atmospheric states are assumed to be constant within the individual PM due to their limited spatial extent. The variation of the UAV position also changes the length of the line of sight, which would result in a change of the GSD. Therefore, the field of view of the sensor is varied within its technical limits so that the GSD reference value (see Table 1) is maintained.

**Figure 7:**
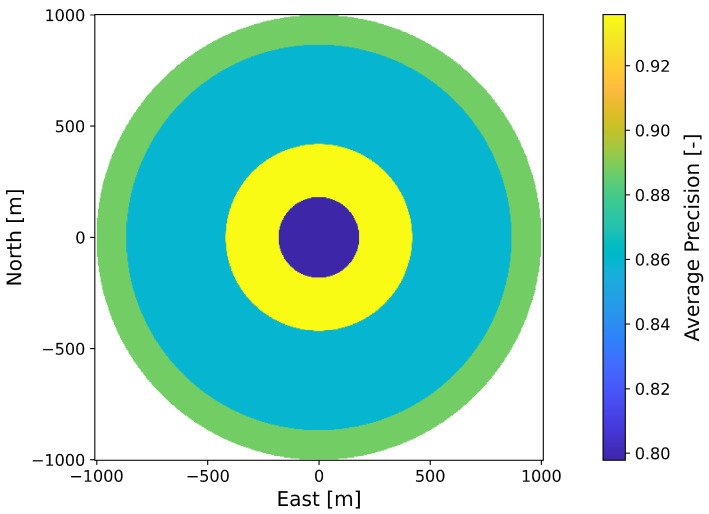
Perception map resulting from the Yolo-SPM sensor performance model.

In the left image of Figure 6, a perception map resulting from the CC sensor performance model is shown as a three-dimensional plot. The north-east plane coincides with the UAV’s plane of motion and the z-direction represents the quantitative value of the predicted detection performance as a measure of the F1-score or the average precision, depending on the applied sensor performance model. The detection performance is color-coded for better illustration. The perception map shown contains areas with a maximum performance of 0.98 (yellow colored area) and a minimum value of 0.83 (blue colored area). If sensor data are acquired in an area of the perception map with a high value, this will result in high predicted detection performance for the applied perception chain. This is indicated in the right graph by a camera symbol in the yellow region. From this plotted sensor position and the corresponding elevation angle α, a high detection performance results. The elevation angle is calculated from the horizontal distance $d_{hor}$ and the altitude above ground $h_{agl}$. A representation of the perception map resulting from the sensor performance model with the YOLOv3 object detector is given in Figure 7.

In contrast to the deep learning-based performance model from Section 2.2.1, the machine learning-based performance model yields a continuous and differentiable profile of the detection performance under variation of the elevation angle. This is due to the mapping of the detection performance by a neural network. In contrast, the deep learning-based performance model yields section-wise constant detection performance, which can be seen in Figure 7.

The concept of perception maps has the advantage that it can be applied to implicit and explicit functions as well as to differentiable (as with the sensor performance model from Section 2.2.2) as well as non-differentiable and discontinuous functions (as with the sensor performance model from Section 2.2.1).

Another decisive advantage is that the maximum value of the detection performance can be determined for each individual sensor footprint from the associated perception map. This maximum value is therefore the upper bound of the detection performance of the respective perception map. The average of the maximum values of all perception maps yields the *maximum average detection performance* for the assigned reconnaissance task. This value is used in Section 3 to validate the achieved detection performance by our trajectory optimization.

### 2.3. Optimal Control for UAV Trajectory Optimization

*Optimal control* is an essential part of the two optimization methods *nonlinear model predictive control* (Section 2.4.2) and *dynamic programming and optimal control* (Section 2.5.2) used in this work for trajectory optimization. For this reason, the theoretical foundations of optimal control for discrete-time systems are presented in this section as far as necessary. The application to continuous-time systems is treated separately in the corresponding section. Nevertheless, the main features of optimal control are identical for both systems.

In general, optimal control aims for determining the control inputs for a dynamical system in such a way that a specific objective function is minimized with respect to system state constraints. With control inputs, the course of the dynamical system state over time can be affected. It is assumed that the evolution of the system state over time is deterministic.

The discrete-time *optimal control problem* (OCP) in its general form can be formulated according to Equation (3):

(3a) $$\begin{array}{cc} \underset{x_{0},u_{0},x_{1},u_{1},\ldots ,u_{N-1},x_{N}}{\mathrm{minimize}}\;J & =E\left(x_{N}\right)+\sum_{k=0}^{N-1}L\left(x_{k},u_{k}\right)\;\;\\ \mathrm{subject}\;\mathrm{to}\;\; & \;\;\;\; \end{array}$$

(3b) $$\begin{array}{cc} x_{k+1}-f\left(x_{k},u_{k}\right) & =0,\;k=0,\ldots ,N-1 \end{array}$$

(3c) $$\begin{array}{cc} x_{k} & \in S_{k},\;\;\;k=0,\ldots ,N \end{array}$$

(3d) $$\begin{array}{cc} u_{k} & \in U_{k},\;\;k=0,\ldots ,N-1 \end{array}$$

(3e) $$\begin{array}{cc} r(x_{0},x_{N}) & =0 \end{array}$$

With $x_{k}$ as the discrete-time state vector of the system, the control input vector $u_{k}$, the discrete time step $k\in N_{0}$, and the time horizon of length *N*. The discrete dynamic system (3b) describes the transition from one state $x_{k}$ at time step *k* to the next state $x_{k+1}$ in the following time step k+1 caused by the control input $u_{k}$. Equation (3a) gives the performance measure of the objective or cost function comprised of the terminal cost $E(x_{N})$ and the time step dependent cost $L(x_{k},u_{k})$, which is additive over time.

The goal of optimal control is to choose the control vector $u_{k}$ in such a way that the cost function J∈R (3a) is minimized for the discrete time steps *k*, under consideration of the constraints (3b) to (3e). The constraints take into account the discrete-time differential Equation (3b) of the UAV’s flight dynamic system, which will be defined by Dubin’s paths in Section 2.5.1. Furthermore, Equation (3c) accounts for system state constraints and Equation (3d) addresses control input constraints on the OCP. Additionally, initial and/or final system state constraints can be specified by Equation (3e).

The feedback control law $\mu_{k}$ (4) maps the system states $x_{k}$ to the control inputs $u_{k}$.

(4) $$\begin{array}{c} u_{k}=\mu_{k}\left(x_{k}\right)\\ \mu_{k}\left(x_{k}\right)\in U_{k}\left(x_{k}\right)\;\forall \;x_{k}\in S_{k} \end{array}$$

A sequence of admissible $\mu_{k}$ composes the control strategy π (5) over all time steps.

(5) $$\begin{array}{c} \pi =\{\mu_{0},\ldots ,\mu_{N-1}\}\in \Pi \\ \Pi =\{\pi =\left(\mu_{0},\ldots ,\mu_{N-1}\right)|\mu_{k}:R^{n_{x}}\to R^{n_{u}}\} \end{array}$$

By applying Equation (3b), a given control strategy π (5) and a specific initial value of the system state vector $x_{0}\in S_{k}$, the state transition (6) can be calculated:

(6) $$x_{k+1}^{\pi ,x_{0}}-f\left(x_{k}^{\pi ,x_{0}},\mu_{k}\left(x_{k}^{\pi ,x_{0}}\right)\right)=0,\;k=0,\ldots ,N-1$$

The optimal control strategy

(7) $$\pi^{*}=\left\{\mu_{0}^{*},\mu_{1}^{*},\ldots ,\mu_{N-1}^{*}\right\}$$

is characterized by minimizing the total cost $J_{\pi^{*}}\left(x_{0}\right)$ (9) of the objective function (8) for a given initial state vector $x_{0}$, satisfying the specified constraints. The total cumulative cost of an admissible control strategy is constrained to be $J_{\pi }<\infty$.

(8) $$\begin{array}{c} J_{\pi }\left(x_{0}\right)=E\left(x_{N}^{\pi ,x_{0}}\right)+\sum_{k=0}^{N-1}L\left(x_{k}^{\pi ,x_{0}},\mu_{k}\left(x_{k}^{\pi ,x_{0}}\right)\right) \end{array}$$

(9) $$\begin{array}{c} J_{\pi^{*}}\left(x_{0}\right)=\min_{\pi \in \Pi }\;J_{\pi }\left(x_{0}\right) \end{array}$$

With the optimization method *dynamic programming and optimal control* (DP&OC), a global optimal reference trajectory is generated with respect to its state space. On the other hand, with the method *nonlinear model predictive control* (NMPC) a locally optimal reference trajectory is obtained for a given starting point and heading. Trajectory optimization with DP&OC and NMPC has already been described in our previous work [13,14]. Both approaches are described in condensed form in this paper to better understand their use and evaluation with the newly developed sensor performance model (Section 2.2.1).

Before discussing the trajectory optimization methods in more detail, we will define some simplifications and constraints that apply to both methods:

- The earth is assumed stationary and flat.
- The earth-fixed coordinate system is considered as an inertial system.
- The influence of wind or turbulence on the motion of the aircraft is neglected.
- The airspeed is predefined and can be considered approximately constant.
- The UAV is assumed to operate at a constant altitude, making the equation of motion for vertical motion obsolete.

Further, it is assumed that the UAV features an autopilot and appropriate sensors on board to recognize and compensate for deviations (e.g., due to wind or turbulence) of the pre-planned reference flight trajectory by itself. For this purpose, with trajectory optimization, it is necessary to provide margins with respect to the flight envelope limits of the UAV in order to remain within the permissible limitations at all times. Moreover, it is assumed that the UAV’s imaging sensor is attached to the UAV by a gimbal, which allows the sensor to be aligned within technical limits independently of the UAV’s orientation. Furthermore, the gimbal dynamics, and thus the sensor alignment, are considered to be significantly faster than the UAV dynamics. Therefore, we do not explicitly consider gimbal dynamics in the remainder of this paper.

### 2.4. Trajectory Optimization with Nonlinear Model Predictive Control

We presented our approach of generating optimized flight trajectories with *nonlinear model predictive control* to enhance detection performance in [13]. It is based on *path planning* (Section 2.4.1) followed by the actual trajectory optimization with *nonlinear model predictive control* in Section 2.4.2 and Section 2.4.3. The next sections provide an overview of the approach to be able to examine the functionality of the sensor-model-based trajectory optimization for time-continuous systems with our newly developed sensor performance model (Section 2.2.1) followed by an evaluation in Section 3. In the following, the continuous-time system is discretized in time by the time step interval Δt for the computation of the optimization. In contrast to DP&OC, the state space remains continuous.

The approach to trajectory optimization can briefly be summarized according to [13]: Path planning is performed for each discretized time step to determine the future evolution of the detection performance along the paths. It is followed by the actual UAV trajectory optimization using NMPC. In this process, a previously determined optimal path acts as the setpoint input of the trajectory optimization. The result is an optimal control input to be applied for one time step. Applying the optimal control inputs for all time steps results in the locally optimal UAV flight trajectory for a given starting position.

It is known from Section 2.2.3 that the detection performance in the UAV’s motion plane resembles a potential field that varies, depending on the environmental state. Therefore, we use the combined approach with the preceding path planning for the following reasons: Compared to classical methods of path planning with potential fields (e.g., [46]), our approach is insensitive to local minima, discontinuities and peaks in the course of the detection performance. Further, in classical path planning with potential fields, planning is carried out from a starting point to a predetermined end point. In our case, there is a given starting point, but the end point is determined by the course of the perception maps and the combined path and trajectory planning and thus, is a priori unknown.

Besides these aspects, the use of NMPC for sensor-model-based trajectory optimization offers the following advantages:

- The solution of the OCP is obtained by closed-loop control. This allows for the compensation of uncertainties between the modeled system dynamics and the real system.
- Model predictive control is one of the few methods to handle hard system state and/or control input constraints [47].
- The course of the setpoints does not need to reproduce the system dynamics exactly.

On the other hand, there are also disadvantages that are necessary to be mentioned:

- A suitable model must be found and modeled in order to be able to reproduce the system dynamics with sufficient accuracy.
- From the nonlinear system dynamics follows a general non-convex optimal control problem, for which only local optimal results can be computed [48].

#### 2.4.1. Fan-Shaped Path Planning

*Path planning* is a part of the combined path and trajectory planning which is outlined in Section 2.4.3 and was first introduced in [13]. The paths fulfill the following two purposes: First, the position and course of the paths serve as set points for trajectory optimization with NMPC. Second, for each path, the expected detection performance is calculated, which would occur if the UAV would fly along the respective path. Here, the detection performance is determined using the time step dependent *perception maps* presented in Section 2.2.3.

The paths form an array originating at the time step-dependent position of the UAV. From the UAV position, the paths spread out in a fan-shape with equal length. The path length $l_{path}$ is determined by Equation (10), with $v_{ref}$ as the predefined setpoint velocity of the UAV, the time step interval Δt from Section 2.1 and $M_{prev}\in N$ as the number of time steps of the preview horizon.

(10) $$l_{path}=v_{ref}\cdot \Delta t\cdot M_{prev}$$

The path planning process can be briefly summarized as follows [13]: Starting from the current UAV position at time step n∈{0,…,T} and T∈N, the detection performance is determined along each path z∈{1,…,Z} with Z∈N for every future time step $m\in \{1,\ldots ,M_{prev}\}$ within the preview horizon. Each time step n+m is assigned a unique sensor footprint by coverage path planning (see Section 2.1) and a corresponding perception map, which maps the course of the local detection performance in the UAV’s motion plane. The position of the UAV and its sensor is determined by the course of the path *z* and the time step dependent distance $d_{path,m}$ in (11) passed by the UAV in the preview horizon.

(11) $$d_{path,m}=v_{ref}\cdot \Delta t\cdot m$$

Finally, the local detection performance $p_{det,n+m,z}$ can be determined from the time step-dependent UAV position and the associated perception map.

The individual detection performances are added up to a weighted *cumulative detection performance* $p_{prev,n+m,z}$ according to Equation (12). Exponential weighting was chosen to strengthen detection performance values closer in time and weaken values further away. The effectiveness of this measure was confirmed by various test cases.

(12) $$p_{prev,n,z}=\frac{1}{M_{prev}}\sum_{m=1}^{M_{prev}}e^{-\frac{m}{M_{prev}}}\cdot p_{det,n+m,z}$$

In Equation (13), the cumulative detection performance values are divided by their maximum value to be normalized before being reused in Section 2.4.3.

(13) $${\tilde{p}}_{prev,n,z}=\frac{p_{prev,n,z}}{\max_{z}\left(p_{prev,n,z}\right)}\;\mathrm{with}\;\max_{z}\left(p_{prev,n,z}\right)\neq 0$$

Here, ${\tilde{p}}_{prev,n,z}\in \left[0,1\right]$ is the *normalized cumulative detection performance* of path *z* and time step *n*. In Table 4, the relevant parameter settings for the path-planning process are summarized.

The shape of the paths approximates the flight behavior of a fixed-wing aircraft. Each path has a fixed curvature, creating evenly distributed fan-shaped curve segments that cover the range between a sharp left turn to a sharp right turn. The number of paths and their curvature define the area in the UAV’s flight direction that is covered by the paths. Whereas the path length determines the number of future time steps and thus the temporal preview horizon.

**Figure 8:**
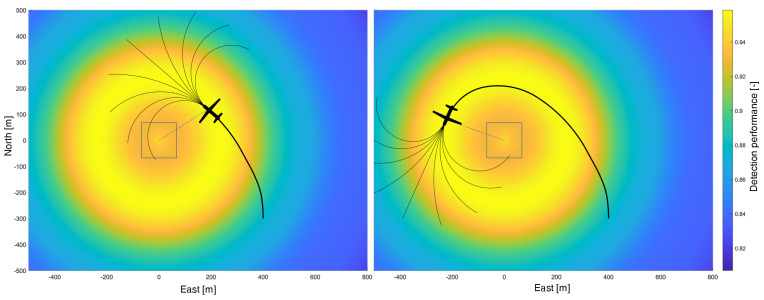
Depiction of the principle of path planning. The fan-shaped path array consists (for representational reasons) of 9 evenly spaced curves (thin black lines). The thick black line is the resulting UAV trajectory from trajectory optimization. The square represents the sensor footprint on the ground. The perception map, which results from atmospheric and topographic conditions is color-coded. Yellow areas mark regions with high detection performance. Adapted from [13].

Figure 8 illustrates the principle of path planning using a stationary sensor footprint as an example. The fan-shaped path array is shown, which is used to determine the future expected detection performance along each individual path at each time step. The thick black line represents the optimized UAV flight trajectory and is the result of the combination of path planning and nonlinear model predictive control, which is described in Section 2.4.3. The perception map results from the atmospheric and topographic conditions and is illustrated as a color-coded potential field. Bright areas mark regions of high detection performance, whereas darker areas map low performances.

#### 2.4.2. Nonlinear Model Predictive Control

In this section, the principles of *nonlinear model predictive control* are discussed. This is in preparation for the combined path and trajectory optimization in the next section.

In Equation (14), the temporal change of the system state for a general continuous-time dynamical system is described by an ordinary differential equation in explicit form.

(14) $$\dot{x}\left(t\right)=F\left(x\left(t\right),u\left(t\right)\right),\;t\in \left[0,T\right]$$

Here, $x\left(t\right)\in R^{n_{x}}$ is the continuous-time state vector of the system, $u\left(t\right)\in R^{n_{u}}$ is the control input vector and *t* is the time. The mapping rule $F:R^{n_{x}}\times R^{n_{u}}\times \left[0,T\right]\to R^{n_{x}}$ describes the variation of the system state over time as a function of the system state itself, the control input and the time. The differential Equation (15) give the nonlinear continuous-time equations of motion for a fixed-wing UAV in the inertial frame of a two-dimensional horizontal plane [13], taking into account the simplifications made in Section 2.3.

(15a) $$\begin{array}{cc} \dot{e}\left(t\right) & =v(t)\cdot \sin(\psi (t)) \end{array}$$

(15b) $$\begin{array}{cc} \dot{n}\left(t\right) & =v(t)\cdot \cos(\psi (t)) \end{array}$$

(15c) $$\begin{array}{cc} \dot{\psi }\left(t\right) & =\frac{g}{v(t)}\cdot \tan\left(\phi \left(t\right)\right) \end{array}$$

(15d) $$\begin{array}{cc} \dot{v}\left(t\right) & =a(t) \end{array}$$

(15e) $$\begin{array}{cc} \dot{\phi }\left(t\right) & =\omega (t) \end{array}$$

(16) $$x\left(t\right)={\left[e\left(t\right),n\left(t\right),\psi \left(t\right),v\left(t\right),\phi \left(t\right)\right]}^{T}$$

The system state vector (16) of the nonlinear dynamic system (15) comprises the north n(t) and east e(t) position, the yaw angle ψ(t), and the horizontal velocity v(t) and roll angle ϕ(t) of the UAV. The gravitational acceleration, which is considered constant, is denoted by *g*. The UAV flies in a two-dimensional horizontal plane at a constant altitude. Therefore, altitude is not considered a state variable. The control input vector (17) consists of the acceleration a(t) of the UAV tangential to the flight path and the roll rate ω(t).

(17) $$u\left(t\right)={\left[a\left(t\right),\omega \left(t\right)\right]}^{T}$$

In order to implement flight envelope limitations for the UAV, state and control restrictions are applied:

(18) $$\begin{array}{c} \left|\phi \left(t\right)\right|\leq \phi_{max} \end{array}$$

(19) $$\begin{array}{c} \left|\omega \left(t\right)\right|\leq \omega_{max} \end{array}$$

(20) $$\begin{array}{c} \left|a\left(t\right)\right|\leq a_{max} \end{array}$$

(21) $$\begin{array}{c} v_{min}\leq v\left(t\right)\leq v_{max} \end{array}$$

With $\phi_{max}$, $\omega_{max}$, $v_{min/max}$ and $a_{max}\in R_{+}$. The parameter settings of the control and state constraints as used for trajectory optimization with NMPC are listed in Table 5.

With nonlinear model predictive control, a discrete-time open-loop optimal control problem is solved periodically for each time step $t_{n}$ with n∈{0,…,T} over a *prediction horizon* N∈N. The first control input $u_{0}^{*}=\mu_{0}^{*}\left(x_{0}\right)$ resulting from the solution of the OCP is applied to the dynamic system (14). In the subsequent time step, the OCP is solved again based on the newly evolved system state.

For this, the continuous-time equations of motion (14) of the UAV are discretized, e.g., using Euler discretization method with the sample time interval Δt, which yields Equation (22b). In addition, the OCP in its general form (3) is slightly adapted for the use with NMPC:

(22a) $$\begin{array}{cc} \underset{x_{0},u_{0},x_{1},u_{1},\ldots ,u_{N-1},x_{N}}{\mathrm{minimize}}\;J & =\sum_{k=0}^{N-1}L\left(x_{n+k},u_{n+k}\right)\\ \mathrm{subject}\;\mathrm{to}\;\; & \;\;\;\;\; \end{array}$$

(22b) $$\begin{array}{cc} x_{n+k+1} & =f(x_{n+k},u_{n+k}) \end{array}$$

(22c) $$\begin{array}{cc} u_{n+k} & \in U_{n+k}\left(x_{n+k}\right) \end{array}$$

(22d) $$\begin{array}{cc} x_{n+k} & \in S_{n+k} \end{array}$$

The transition from state $x_{n+k}$ to the subsequent state $x_{n+k+1}$ is described in Equation (22b). Here, *n* is the current time step and k∈{0,…,N} is the number of time steps ahead in the prediction horizon.

Equation (23) represents the objective function of (22a) in the common quadratic form. The vectors $x_{m}^{ref}$ (26) and $u_{m}^{ref}$ (27) describe time step-specific setpoints for the system state and for the control input, respectively. The index *m* (28) is a placeholder for the specific time step.

(23) $$\begin{array}{cc} L(x_{n+k},u_{n+k}) & ={\tilde{x}}_{n+k}^{T}\;Q\;{\tilde{x}}_{n+k}+{\tilde{u}}_{n+k}^{T}\;R\;{\tilde{u}}_{n+k} \end{array}$$

(24) $$\begin{array}{cc} {\tilde{x}}_{m} & =x_{m}-x_{m}^{ref} \end{array}$$

(25) $$\begin{array}{cc} {\tilde{u}}_{m} & =u_{m}-u_{m}^{ref} \end{array}$$

(26) $$\begin{array}{cc} x_{m}^{ref} & ={\left[e_{m}^{ref},n_{m}^{ref},\psi_{m}^{ref},v_{m}^{ref},\phi_{m}^{ref}\right]}^{T} \end{array}$$

(27) $$\begin{array}{cc} u_{m}^{ref} & ={\left[a_{m}^{ref},\omega_{m}^{ref}\right]}^{T} \end{array}$$

(28) $$\begin{array}{cc} m & =n+k \end{array}$$

*Q* and *R* in (23) are positive definite symmetric weighting matrices with their values defined in Table 5. These matrices determine which components of the system state vector and the control vector are considered for the calculation of the objective function and the weighting of these components.

After adapting the time steps from *k* to n+k for Equations (4) to (8), the minimum total cost $J_{\pi^{*}}\left(x_{n}\right)$ (29) is obtained from OCP (22a) over the prediction horizon at time step *n*.

(29) $$J_{\pi^{*}}\left(x_{n}\right)=\min_{\pi \in \Pi }\;J_{\pi }\left(x_{n}\right)$$

The algorithm for the calculation of the nonlinear model predictive control is performed for each sampling time $t_{n}$ of the optimization problem as follows (adapted from [49]):

1. The current system state $x_{n}$ at time $t_{n}$ is measured.
2. The optimal control problem (22) is solved for the quadratic objective function (23) and the setpoint values $x_{n+k}^{ref}$ and $u_{n+k}^{ref}$. The result is the optimal control strategy $\pi^{*}\left(x_{n}\right)$ with respect to the current state $x_{n}$.
3. From the optimal control strategy $\pi^{*}\left(x_{n}\right)$, the initial control input $u_{n}^{*}=\mu_{n}^{*}\left(x_{n}\right)$ is applied to the dynamical system for the duration of one time step Δt.
4. At the end of the time step, the updated system state $x_{n+1}$ is measured at time $t_{n+1}$.
5. The NMPC algorithm starts again at point 1 with the updated system state and continues until all time steps $t_{n}$ have been processed.

The calculation of the solution for NMPC can be carried out, for example, by utilizing single shooting [50] or multiple shooting methods [51,52] or by sequential quadratic programming [53]. For studies on the stability, robustness and optimality of nonlinear model predictive control, we refer to [54,55]. For a more detailed description of nonlinear model predictive control in general, we refer the reader to [47,56,57].

#### 2.4.3. Combining Path Planning and NMPC for Trajectory Optimization

After introducing the essential aspects of path planning and NMPC, they are combined as described in [13] to calculate the optimal control input at each time step $t_{n}$. This optimal control input incorporates the optimal weighted ratio of high expected detection performance and low cost from the OCP. The approach is executed for each time step $t_{n}$ as follows:

For the current time step *n* the position and heading of the UAV are obtained. From this, the positioning of the path array (see Section 2.4.1) is determined. For each path in the path array, the cumulative detection performance $p_{prev,t,z}$ along the path is calculated. In the next step, the cumulative detection performance values are normalized as stated in Equation (13) yielding the path and time step dependent normalized cumulative detection performance ${\tilde{p}}_{prev,t,z}\in \left[0,1\right]$.

This process is performed in a similar way for the calculation of the cost function using NMPC. For this, each path *z* of the path array serves as a setpoint yielding the time step-specific reference values $n_{n+k}^{ref}$, $e_{n+k}^{ref}$ and $\psi_{n+k}^{ref}$ along the prediction horizon *N* for the OCP. This leads to the path and time step dependent minimum total cost $J_{\pi }\left(x_{n},z\right)$ according to Equation (22a). In the next step, the minimum total cost values are scaled by their largest value to be normalized according to (30).

(30) $${\tilde{J}}_{\pi }\left(x_{n},z\right)=\frac{J_{\pi }\left(x_{n},z\right)}{\max_{z}\left(J_{\pi }\left(x_{n},z\right)\right)}\;\mathrm{with}\;\max_{z}\left(J_{\pi }\left(x_{n},z\right)\right)\neq 0$$

The final step in the combined path planning and trajectory optimization is to determine the optimal path from the path array that combines the best detection performance with the lowest total cost resulting from the OCP at time step *n*. For this, the normalized minimum total cost ${\tilde{J}}_{\pi }\left(x_{n},z\right)\in \left[0,1\right]$ and the normalized cumulative detection performance ${\tilde{p}}_{prev,t,z}$ are weighted by γ∈[0,1] (see Table 6) and processed according to Equation (31).

(31) $$c_{min,n}=\min_{z}\left(\left(1-\gamma \right)\cdot {\tilde{J}}_{\pi }\left(x_{n},z\right)-\gamma \cdot {\tilde{p}}_{det,n,z}\right)$$

This results in a time step-dependent combined minimum cost $c_{min,n}$ of the detection performance and the OCP. The minimum cost $c_{min,n}$ relates to the optimal path *z* that incorporates the best combination of benefit and effort. Furthermore, this optimal path is the set point for the NMPC optimization and yields the optimal control input $u_{n}^{*}$ for the next time step.

From the processing of all time steps *n*, an optimal control strategy $\pi^{*}\left(x_{0}\right)$ (7) results with respect to the starting point $x_{0}$. This control strategy determines the spatio-temporal positioning of the UAV and thus the (optimal) flight trajectory. Furthermore, by this trajectory, the final detection performance is determined, which would arise from the application of this trajectory.

### 2.5. Trajectory Optimization with Dynamic Programming

*Dynamic programming and optimal control* is an optimization method that can be used for generating optimal sensor-model-based UAV flight trajectories for discrete-time and discrete-value systems. This approach was first described in [14] where the state transitions were modeled in a simplified way by line segments, which were restricted in length and change of direction. To achieve a smooth trajectory the line segments were approximated by splines. In this paper, we present a new approach in which the state transition in DP&OC is realized using *Dubins paths*, which is described in the next section. This allows the explicit limitation of the admissible curve radius to meet g-load constraints, which could not be realized with our previous approach.

#### 2.5.1. Dubins Path

*Dubins path planning* was first outlined by Dubins [58] and describes a method to identify the shortest path connecting a start configuration with a goal configuration in a two-dimensional plane under curvature constraint. The configuration is the position of the start or goal point in the plane of motion and the associated direction (heading) of the velocity vector. In this work, we describe for the first time the use of Dubins paths for modeling the discrete state transitions in sensor-model-based trajectory optimization with DP&OC.

Dubins paths are used in this work to model the state transitions for the discrete optimization method of dynamic programming. This is motivated by two major advantages of Dubins paths: the paths are curvature constrained, taking the flight envelope limit for the allowable acceleration into account. Furthermore, the principle of the Dubins path results in the shortest (flight) path between two configurations, which ultimately minimizes the flight duration.

For modeling the trajectory of a fixed-wing UAV using Dubins paths, a forward velocity v(t)>0 must be assumed. This excludes backward motion, which distinguishes the Dubins path from the principle of the Reeds-Shepp curve [59]. The Dubins path is a commonly used method for simplified modeling of time-optimal UAV trajectories with respect to curvature constraints. Numerous application examples can be found in the literature, for instance in [60,61,62].

In the following, the basic principles of Dubins path planning are presented before they are combined into a global optimal trajectory of concatenated path segments using dynamic programming. For this, the following two criteria must be satisfied ([63] p. 880):

- The velocity *v* of the UAV must be set constant.
- The maximum permissible roll angle $\phi_{max}$ has to be defined.

The Dubins path is generated from the set of motion primitives {L,S,R}, where *L* is a left-hand curve of maximum curvature, *R* is a right-hand curve of maximum curvature, and *S* is a straight line segment. Equations (32) to (34) describe the motion of a UAV that moves in the plane according to the criteria of Dubins path planning.

(32) $$\begin{array}{cc} \dot{n}\left(t\right) & =vs.\cdot \cos(\psi (t)) \end{array}$$

(33) $$\begin{array}{cc} \dot{e}\left(t\right) & =vs.\cdot \sin(\psi (t)) \end{array}$$

(34) $$\begin{array}{cc} \dot{\psi }\left(t\right) & =\frac{g}{v}\cdot \tan\left(\phi \left(t\right)\right) \end{array}$$

$$\begin{array}{cc} \left|\phi (t)\right| & \leq \phi_{max} \end{array}$$

With n(t) and e(t) as position coordinates of the UAV in the earth-fixed coordinate system and the discrete control input u(t)=ϕ(t) with u(t)∈U={(l,0,l), (l,0,r), (r,0,l), (r,0,r), (l,r,l), (r,l,r)} and $l=-\phi_{max}$ as well as $r=\phi_{max}$. Further, $\dot{\psi }\left(t\right)$ is the turn rate as a function of the roll angle ϕ(t), the gravitational acceleration *g*, and the constant flight path velocity *v*. As a result of the discrete control inputs u(t), the roll angle ϕ(t) and roll rate $\dot{\phi }\left(t\right)$ change abruptly during the transition between the motion primitives. The trajectory is therefore not $C^{2}$-continuous.

For the motion primitives *L* and *R*, Equation (35) gives the relationship between the velocity *v*, the maximum roll angle $\phi_{max}$, and the resulting minimal curve radius $r_{min}$, which is indirectly proportional to the maximum path curvature $\kappa_{max}$. Table 7 provides a summary of the corresponding parameter settings.

(35) $$\kappa_{max}=\frac{1}{r_{min}}=\frac{g\cdot \tan(\phi_{max})}{v^{2}}\;\mathrm{with}\;vs.\neq 0$$

Equation (36) describes the arc length *s* of the flight path from a start configuration *a* to a goal configuration *b*. The arc length will be reused in the next section as an evaluation criterion for trajectory optimization.

(36) $$s=\int_{a}^{b}\sqrt{\dot{n}{\left(t\right)}^{2}+\dot{e}{\left(t\right)}^{2}}\;dt$$

**Figure 9:**
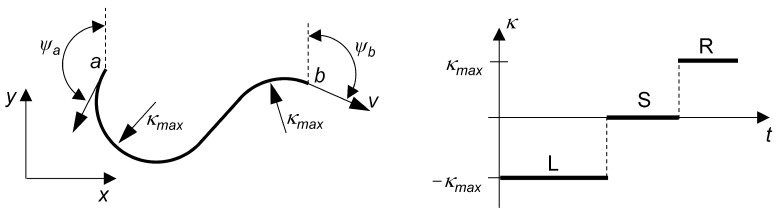
Example of a Dubins path from the start configuration *a* to the goal configuration *b* defined by a specific set of motion primitives *L*, *S* and *R* (**left**). The associated curvature profile of the Dubins path is plotted on the (**right**).

Figure 9 illustrates an example of connecting two points *a* and *b* with given yaw angles $\psi_{a}$ and $\psi_{b}$ by a Dubins path. The control input u(t) is composed of a specific configuration of the section-wise constant motion primitives *L*, *S* and *R* at a constant flight velocity *v*. For the solution of the Dubins path planning problem, we refer to the literature, for instance [64,65].

#### 2.5.2. Dynamic Programming and Optimal Control

With *dynamic programming and optimal control* a discrete *optimal control problem*, which was described in Section 2.3, can be solved. DP&OC enables the computation of global optimal reference trajectories with respect to discretization. In this work, DP&OC is used to generate UAV flight trajectories from Dubins path segments. A key advantage of dynamic programming is that non-differentiable system dynamics can be used, such as with the section-wise constant roll angle input in Dubins path planning. In the final step, the trajectory from Dubins path planning is smoothed to meet the requirement from Section 2 for continuous curvature.

Dynamic programming and optimal control are based on the *principle of optimality* [66] and is a method to solve a discrete-time, discrete-value OCP. It was developed in the 1950s, in particular by Bellman [66]. According to [67], the principle of optimality can be described in a simplified way that every subtrajectory of an optimal trajectory is an optimal trajectory itself. It can be expressed mathematically as follows:

Let $\pi^{*}$ be the optimal control strategy for an OCP, then $\left\{\mu_{i}^{*},\mu_{i+1}^{*},\ldots ,\mu_{N-1}^{*}\right\}$ is the optimal control strategy for the subproblem from time *l* to the final time step *N* that minimizes the cost of the objective function $J_{\pi^{*}}\left(x_{l}\right)$, with

(37) $$J_{\pi^{*}}\left(x_{l}\right)=\min_{\pi \in \Pi }\left\{E\left(x_{N}^{\pi ,x_{0}}\right)+\sum_{k=l}^{N-1}L\left(x_{k}^{\pi ,x_{0}},\mu_{k}\left(x_{k}^{\pi ,x_{0}}\right)\right)\right\}.$$

**Figure 10:**
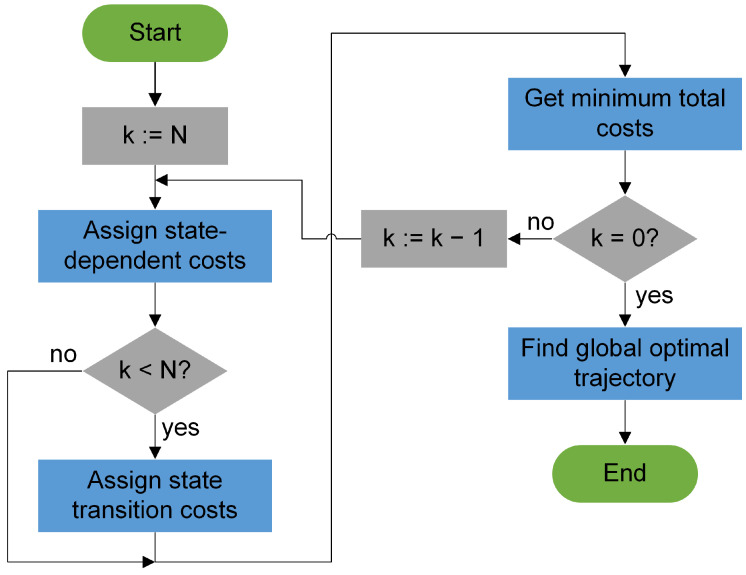
Flow chart of the DP&OC process for time steps k=0,…, *N*.

Thus, optimization in dynamic programming starts at the final time step k=N and proceeds backward to the first time step k=0. The DP&OC process is described in the following and depicted graphically in Figure 10.

For each time step *k* the states in Equation (38) are assigned and to the following time step k+1 the states in Equation (39) are allocated.

(38) $$\begin{array}{cc} x_{k}^{i},\;i & =1,\ldots ,n_{k}\;\mathrm{with}\;n_{k}\in N\;\mathrm{in}\;k\in \left\{0,\ldots ,N\right\} \end{array}$$

(39) $$\begin{array}{cc} x_{k+1}^{j},\;j & =1,\ldots ,n_{k+1}\;\mathrm{with}\;n_{k+1}\in N\;\mathrm{in}\;k\in \left\{0,\ldots ,N-1\right\} \end{array}$$

(40) $$\begin{array}{cc} x_{k}^{i} & ={\left[n_{k}^{i},e_{k}^{i},\psi_{k}^{i}\right]}^{T} \end{array}$$

Here, $x_{k}$ is the state vector at time step *k*. *i* and *j* are time step-dependent indices for specific state characteristics. The state vector (40) of the discrete-time and discrete-value system is composed of the position coordinates $n_{k}^{i}$ and $e_{k}^{i}$ of the UAV and its yaw angle $\psi_{k}^{i}$. The number of different state characteristics $m_{state,n}$ (41) per time step results from the number of north $m_{north,n}$ and east $m_{east,n}$ positions, as well as the number of different yaw angles $m_{\psi ,n}$.

(41) $$m_{state,n}=m_{north,n}\cdot m_{east,n}\cdot m_{\psi ,n}$$

The number of different north and east positions arises from the grid of the perception map, whereas the number of yaw angles is predefined and can be found in Table 8.

The spatial discretization in the north and east directions is performed on a grid with the equidistant spacing of $s_{min}$ in Equation (44). The set of states at time step k∈{0,…,N} is defined by $S_{k}=\left\{x_{k}^{i},\ldots ,x_{k}^{n_{k}}\right\}$. Each pair of states $x_{k}^{i}$ in time step *k* and $x_{k+1}^{j}$ in time step k+1 can be associated with a *state transition cost* given in Equation (42).

(42) $$c_{trans,k}^{ij}=c_{trans}\left(x_{k}^{i},x_{k+1}^{j}\right)$$

The state transition cost represents the length of the Dubins path calculated in Equation (36) from the starting configuration $x_{k}^{i}$ to the goal configuration $x_{k+1}^{j}$ and is stated in the following Equation (43):

(43) $$\begin{array}{c} c_{trans,k}^{ij}=\left\{\begin{array}{cc} \infty & \mathrm{if}\;s_{k}^{ij}<s_{min}\\ \frac{1}{s_{max}-s_{min}}\cdot s_{k}^{ij}+\frac{-s_{max}}{s_{max}-s_{min}} & \mathrm{if}\;s_{min}\leq s_{k}^{ij}\leq s_{max}\\ \infty & \mathrm{if}\;s_{k}^{ij}>s_{max} \end{array}\right. \end{array}$$

(44) $$\begin{array}{c} s_{min}=\Delta t\cdot v \end{array}$$

(45) $$\begin{array}{c} s_{max}=r_{min}\cdot \pi \end{array}$$

Here, $s_{min}$ in (44) is the minimum distance the UAV can travel within one time step Δt at the predefined speed *v*. On the other hand, $s_{max}$ in (45) is defined as the maximum permissible path length which allows a half circle to be flown. Equation (43) shows that short path lengths result in low transition costs and large path lengths are penalized. Dubins path lengths shorter than $s_{min}$ are impossible, and path lengths greater than $s_{max}$ are undesirable and therefore assigned an infinite cost. Between $s_{min}$ and $s_{max}$, the transition costs are $c_{trans,k}^{ij}\in \left[0,1\right]$.

Furthermore, *state-dependent costs* in (46) are assigned to each system state $x_{k}^{i}$ for the time step *k*.

(46) $$c_{state,k}^{i}=c_{state}\left(x_{k}^{i}\right)$$

The state-dependent cost corresponds to the local detection performance at position $n_{k}^{i}$ and $e_{k}^{i}$ and arises from the time step-dependent perception map (see Section 2.2.3). The detection performance, and therefore the state-dependent cost, is per definition $c_{state,k}^{i}\in \left[0,1\right]$.

By applying the *state transition costs* (42) and the *state-dependent costs* (46) to the general *objective function* (3a), the cost (47) for the last time step k=N is obtained.

(47) $$J\left(x_{N}^{i}\right)=E\left(x_{N}\right)=c_{state,N}^{i}\;\forall \;x_{N}^{i}\in S_{N}$$

Equation (48) yields the *minimum total costs* for the time steps k=0,…,N−1 using the principle of optimality. These total costs result from the sum of the current state-dependent cost, the path cost to the subsequent state and the minimum total cost from this subsequent state to the final state. $p_{w}$ is a factor to weigh the state-dependent costs against the state transition costs. A high weighting factor emphasizes an increase in detection performance, with the caveat that this may increase the length of the trajectory. A low weighting factor favors a shorter trajectory, which reduces the reconnaissance time. However, this may also lead to a deterioration of the resulting detection performance.

(48) $$\begin{array}{c} J\left(x_{k}^{i}\right)=L\left(x_{k},u_{k}\right)=\min_{j=0,\ldots ,n_{k+1}}\left\{p_{w}\cdot c_{state,k}^{i}+\left(1-p_{w}\right)\cdot c_{trans,k}^{ij}+J\left(x_{k+1}^{j}\right)\right\} \end{array}$$

$$\begin{array}{c} \forall \;x_{k}^{i}\in S_{k}\;,\;k=0,\ldots ,N-1\\ p_{w}\in \left[0,1\right] \end{array}$$

**Figure 11:**
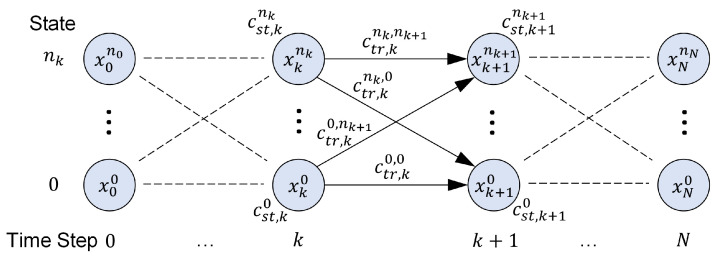
Illustration of the states and the state transitions in an acyclic graph. Circles represent the states $0,\ldots ,n_{k}$ in the individual time steps 0,…,N. The arrows represent the state transitions between two states. As an example, the state-dependent costs $c_{st,k}^{i}$ and the state transition costs $c_{tr,k}^{ij}$ are plotted from time step *k* to k+1.

In Figure 11, the system states (circles), the state-dependent costs (index *st*) and the state transition costs (index *tr*) are exemplarily plotted for two time steps in an acyclic graph.

From the backward calculation and the principle of the optimality follows that each state in the time steps k=0,…,N−1 has a dedicated optimal subsequent state, which combines the minimum total costs of all optimal subsequent states. Therefore, each state is the starting point of an optimal subtrajectory. The iterative continuation of the calculation of the optimal subtrajectory results in the optimal trajectory for a specific initial state. Based on the minimum total cost, an *optimal control*

(49) $$u_{k}^{*}\left(x_{k}\right)=\mu_{k}^{*}\left(x_{k}\right)=\underset{u_{k}\in U_{k}\left(x_{k}\right)}{\arg\;\min\;}\left\{L\left(x_{k},u_{k}\right)+J_{k+1}\left(f\left(x_{k},u_{k}\right)\right)\right\}$$

for each system state $x_{k}$ can be determined [67].

The *global optimal trajectory* can be found by comparing the total cost $J(x_{0}^{i})$ of all associated initial states $x_{0}^{i}$ and identifying the *global minimum total cost* $J(x_{0}^{*})$. The initial state $x_{0}^{*}$ is thus the starting point of the global optimal trajectory with respect to the discretization.

A major disadvantage of DP&OC is that the discretization of the state space increases the computational cost quadratically to the number of system states $x_{k}$. Bellman coined the term “the curse of dimensionality” [66] for this. In order to keep the computation time within acceptable limits, an appropriate discretization of the state space is necessary.

For a more complete description of the dynamic programming algorithms, we refer to the work of Bellman [66] and Bertsekas [67].

#### 2.5.3. Dubins Path Segments Smoothing

In the final step, the trajectory, which is composed of concatenated Dubins path segments, is smoothed. This is to achieve a continuous roll angle transition along the entire trajectory, as required in Section 2. The smoothing procedure is performed by *nonlinear model predictive control* as presented in Section 2.4.2. In this case, the Dubins path segments serve as the setpoint input providing north $n^{ref}$ and east $e^{ref}$ position as well as the yaw angle $\psi^{ref}$ for the optimization. The result is a $C^{2}$-continuous flyable UAV trajectory that satisfies specific flight dynamic constraints, e.g., roll rate, roll angle and velocity limitations.

### 2.6. Benchmark Trajectories

Benchmark trajectories will be used as a baseline to validate the sensor-model-based trajectory optimization. For this purpose, the detection performance resulting from the benchmark trajectory and the optimized trajectory from Section 2.4 and Section 2.5, respectively, will be determined in the next section. By comparing the resulting detection performances, our trajectory optimization approach will be quantitatively validated.

The benchmark trajectories used in the following are based on common loitering patterns used in aviation. It is assumed that these trajectories are either generated automatically by a flight management system on board the UAV or are determined by a UAV operator. In both cases, the planning is carried out without the knowledge or consideration of the sensor performance models from Section 2.2.

The following three loitering patterns are used as benchmark trajectories in this work:

- Circle pattern.
- Racetrack pattern.
- Figure-8 pattern.

**Figure 12:**
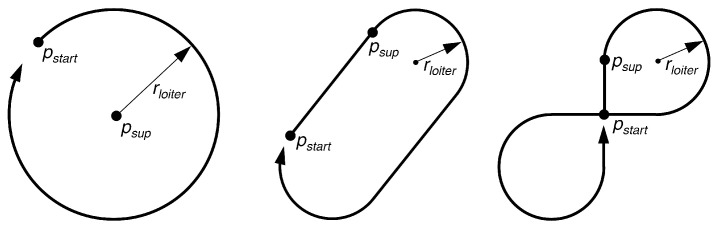
Illustration of the benchmark trajectories Circle (**left**), Racetrack (**center**) and Figure-8 (**right**). Additionally, the starting point $p_{start}$, the support point $p_{sup}$ and the path direction are sketched. The radius $r_{loiter}$ is predefined or results from the design.

All three patterns have in common that they are made up of an easy-to-model geometry and consist of a closed set of lines. Thus, each pattern can be passed through an unlimited number of times. The shape of the benchmark trajectories in our work, which are depicted in Figure 12, is specified by two points, direction information, and radius, where required. These patterns were chosen because they offer different shape characteristics, for example, a constant path curvature for the Circle pattern or sections of straight lines with the Racetrack and Figure-8 patterns.

### 2.7. Implementation

The implementation of the NMPC functionality was carried out in C++, whereas the other parts of the program, such as the program control, the coverage path planning, the calculation of the perception maps and the evaluation were implemented in Python. The communication between the C++ process and the Python modules was realized using ROS 2 [68]. In contrast, for the calculation of the trajectories with DP&OC all necessary program modules were realized in Python.

## 3. Results

We validate our approach to sensor-model-based trajectory optimization by planning optimized reference trajectories for a simulated *route reconnaissance scenario* (Section 3.2) and an *area reconnaissance scenario* (Section 3.3) for vehicle detection with a fixed-wing UAV. With the validation, we aim to demonstrate the ability of our approach to increase the detection performance obtained by the reference trajectory compared to the detection performance achieved by a benchmark trajectory. Furthermore, we compare the detection performance with the theoretical maximum average detection performance, which can be determined by the perception maps (from Section 2.2.3) and acts as an upper bound.

With the route reconnaissance scenario, vehicle detection is to be conducted along a road whereas the route was defined in advance. In the area reconnaissance scenario, vehicles are to be reconnoitered within a predefined area. Coverage path planning determines the spatial and temporal positioning of the sensor footprint along the route or within the area. This task was performed automatically in advance and is not described in detail here.

In the following, the sensor performance model that represents the perception chain including the *YOLOv3* object detector will be referred to as “Yolo-SPM”. Correspondingly, the sensor performance model with the *classification cascade* object classifier is referred to as “CC-SPM”.

### 3.1. Validation Process and General Specifications

The validation process proceeds as follows: With the NMPC trajectory optimization, 12 simulations are performed, each for the route reconnaissance scenario and the area reconnaissance scenario. In each simulation, a benchmark trajectory is defined and a reference trajectory is computed using our approach with NMPC optimization. Here, it is defined that the starting point and the starting heading for both trajectories are identical in order to be able to compare the detection result afterward. Six of the 12 simulations are performed with the CC-SPM and the other six with the Yolo-SPM. The six simulations comprise two different configurations of each of the three different benchmark trajectories (Circle, Racetrack and Figure-8).

For trajectory optimization with DP&OC, two simulations are performed for the route reconnaissance scenario and two for the area reconnaissance scenario. In each case, one simulation is carried out with the CC-SPM sensor performance model and the other one utilizing Yolo-SPM. The result in each case is a global optimal reference trajectory whose expected detection performance is compared with the theoretically maximum average detection performance.

For the NMPC-optimized reference trajectory and the benchmark trajectory, which is only used for the NMPC optimization as a comparison, the sensor footprint velocities are constant (compare Table 1) and equal. Since the UAV velocities are also constant, both the reference and the benchmark trajectories have identical trajectory lengths, resulting in the same minimal reconnaissance duration.

In contrast, the length of the DP&OC-optimized reference trajectory is longer since it is based on a cartesian grid with equidistant spacing. As the UAV velocity is constant and consistent with the other trajectories, the flight time increases. Additionally, because the UAV trajectory is also matched to the sensor movement, the sensor footprint velocity must be dynamically slowed down, however, this will not be discussed in detail in this paper.

The following is assumed for the execution of the simulation: The route or area to be reconnoitered is defined and known a priori. Coverage path planning has already been carried out and is identical for the reference trajectory as well as for the benchmark trajectory. It is assumed that the benchmark trajectories are set by a UAV operator or a flight management system on board the UAV, without the knowledge or consideration of the corresponding sensor performance model. However, the expected detection performance for both trajectories is determined based on the same performance model. This is necessary to be able to compare the detection performance results with each other.

### 3.2. Route Reconnaissance Scenario

**Figure 13:**
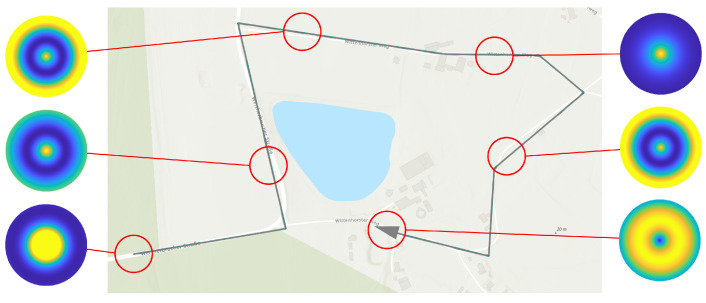
Illustration of the route reconnaissance scenario. The green line marks the reconnaissance route, supplemented by several perception maps resulting from the CC-SPM performance model. The color-coding of the different perception maps corresponds to the predicted detection performance. Light colors represent high performance values, while darker colors correlate with lower values.

With the route reconnaissance scenario, the course of the route was designed in such a way that the topographic conditions included both rural (meadow, vegetation, water) and urban regions (roads, buildings). In addition, the routing should contain several changes of direction to show the ability of the reference trajectory for adaptation. The route has a length of about 2.2 km. The arrow marks the direction in which the reconnaissance task is conducted. Figure 13 shows the reconnaissance route (green line) and provides examples of individual perception maps to give the reader an impression of their different characteristics. Six perception maps of the performance model CC-SPM are depicted (not to scale), which would result in the respective footprint position.

The illustration of the different perception maps is intended to emphasize that the regions with high detection performance can vary significantly from map to map. For example, in the top left perception map, the area of high detection performance is far from the center of the map and thus far from the center of the sensor footprint. In contrast, in the lower left perception map, the area of high performance is concentrated near its center. In the upper right map, the area of high performance is even more localized. To achieve a high overall detection performance for the reconnaissance task, the trajectory optimization has to calculate a reference trajectory that ideally passes only through these areas of high performance, while taking into account additional constraints such as roll angle and roll rate limitations of the UAV.

The perception map resulting from the Yolo-SPM model is consistent across the route and is displayed in Figure 7. It can be seen that the course of the detection performance assumes section-wise constant values and does not have a continuously differentiable characteristic like the perception maps from the CC-SPM.

#### 3.2.1. NMPC Trajectory Optimization

Table 9 lists the *atmospheric states* for the CC-SPM sensor performance model. It is considered that these are determined by mission planning (time of day, month) and the local weather conditions in the reconnaissance area. Due to the localized extent of the reconnaissance area, these are assumed to be constant during the actual reconnaissance operation.

**Figure 14:**
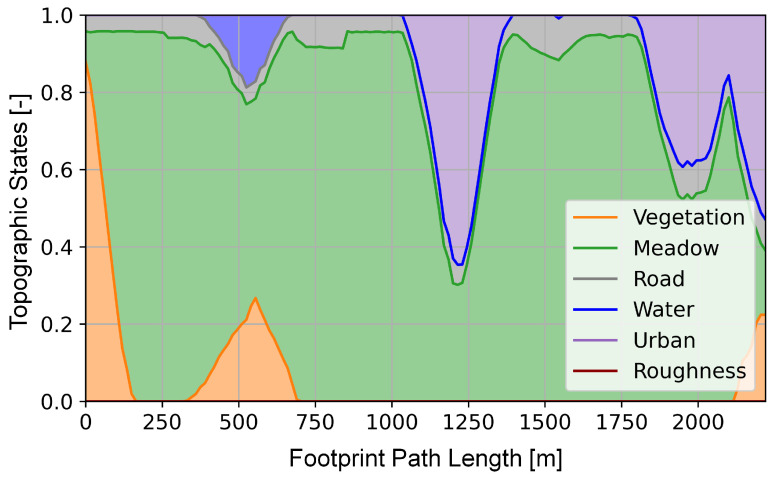
Topographic states resulting from the route reconnaissance scenario.

The *topographic states* are depicted in Figure 14. They result from the content of the sensor footprints along the reconnaissance route and were determined using a geographic information system.

**Figure 15:**
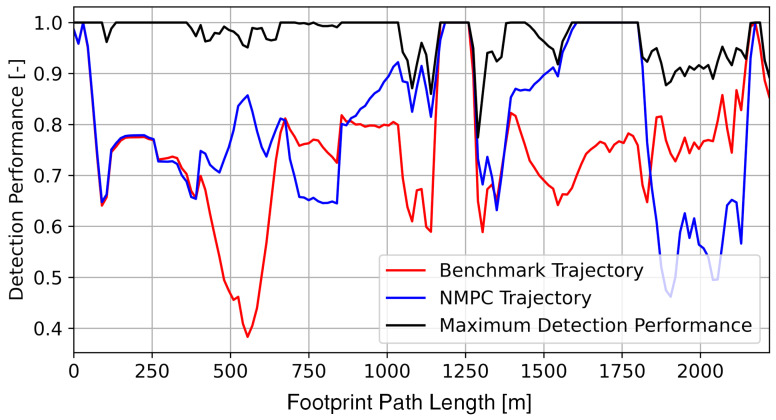
Illustration of the detection performances for the NMPC and benchmark trajectory with respect to the sensor footprint path length. The black line marks the theoretical maximum detection performance as an upper bound.

**Figure 16:**
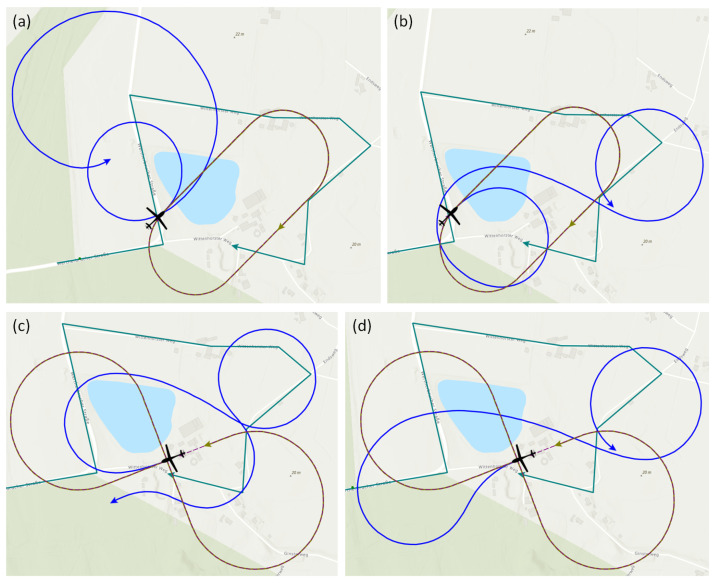
Trajectory optimization for the route reconnaissance scenario with sensor performance model CC-SPM in (**a**,**c**) and with Yolo-SPM in (**b**,**d**). The blue line indicates the NMPC-optimized reference trajectory and the light green line represents the benchmark trajectory. The starting points of both trajectories are identical and marked by a black aircraft symbol. In (**a**,**b**), the Racetrack benchmark pattern is displayed, whereas in (**c**,**d**), the Figure-8 pattern is applied.

The variation of the topographic states along the sensor footprint path results in a highly dynamic change of the perception maps as depicted in Figure 13. The detection performance results from the sensor performance model under the influence of atmospheric, topographic and photographic conditions. The detection performance profile in Figure 15 belongs to the route reconnaissance scenario (a) in Figure 16. The theoretical maximum detection performance is indicated as a black line and acts as an upper bound.

The *atmospheric states* for the Yolo-SPM performance model are listed in Table 10. This performance model does not require any additional topographic conditions to determine the detection performance. For this reason and the assumption that the atmospheric conditions in the reconnaissance area are constant, it follows that the perception maps (see Figure 7) of the individual sensor footprints are all identical.

To give the reader an idea of the trajectory optimization results, Figure 16 shows the reference trajectory (blue line) and the benchmark trajectory (green line) for different simulation settings. Plots (a) and (b) show the Racetrack pattern, whereas (c) and (d) display the Figure-8 pattern. The reference trajectories in plots (a) and (c) were optimized for the CC-SPM model and the Yolo-SPM-optimized trajectories are given in (b) and (d).

**Figure 17:**
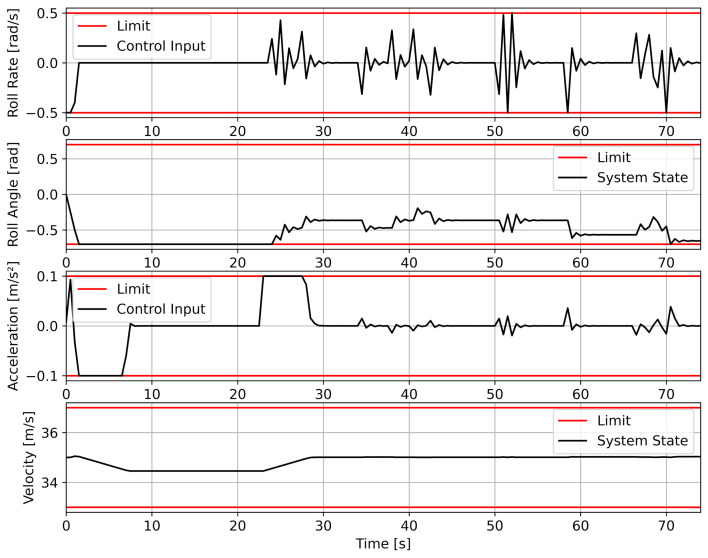
Illustration of the control inputs “roll rate” and “acceleration” for route reconnaissance with CC-SPM, plotted with respect to the flight duration. The control inputs yield changes in the system states “velocity” and “roll angle”. Shown also are the predefined limitations.

In Figure 17, “roll rate” and “acceleration” of the control input vector (17) are plotted, which belong to the experiment sample of the route reconnaissance scenario (a) with CC-SPM in Figure 16. It reveals that the flight dynamic limitations, specified in Table 5, are maintained. The control inputs lead to changes in the system states “velocity” and “roll angle”.

The simulation results for the route reconnaissance scenario with NMPC-optimized reference trajectory and benchmark trajectory are summarized in Table 11. The *maximum average detection performance* is determined from the maximum values of each perception map, which is graphically represented by the upper bound (black line) in Figure 15. Since the coverage path planning and the sensor performance model are identical for the NMPC-optimized trajectory and the benchmark trajectory, the maximum detection performance values are also identical. The *average detection performance* is calculated from the average of each of the six simulations with the CC-SPM or the Yolo-SPM performance model. With the NMPC-optimized reference trajectory utilizing the CC-SPM model, an average increase in detection performance of 4.46% is achieved. Additionally, the NMPC-optimized reference trajectory with the Yolo-SPM, an enhancement of 4.90% is obtained. The length of the flight trajectory is about 2.6 km and approximately identical for both NMPC and benchmark trajectories.

#### 3.2.2. DP&OC Trajectory Optimization

**Figure 18:**
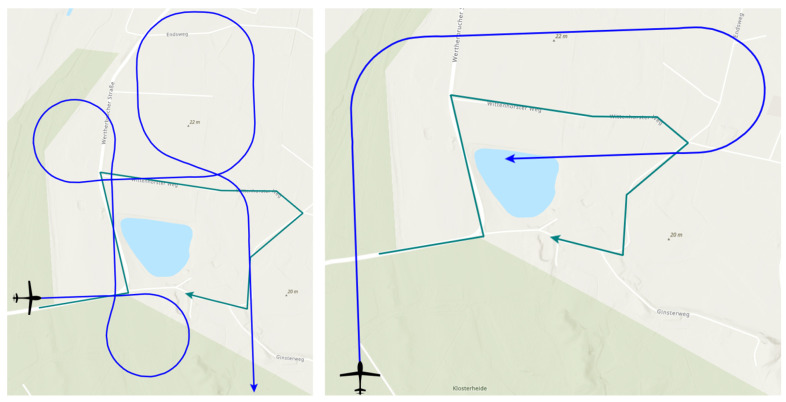
Depiction of the DP&OC-optimized trajectories for the route reconnaissance scenario with sensor performance models CC-SPM (**left**) and Yolo-SPM (**right**). The blue line marks the UAV flight trajectory and the green line maps the sensor footprint on the ground.

By utilizing DPOC optimization, global optimal reference trajectories are generated, thus eliminating the need for a direct comparison with a benchmark trajectory. It follows that only two simulations were performed for route reconnaissance: one using the CC-SPM performance model (Figure 18, left plot) and one using the Yolo-SPM model (Figure 18, right plot).

The results of the simulation are summarized in Table 12. The values of the *maximum average detection performance* are identical to those in Table 11 from the previous section. It can be seen that the trajectory optimization with the Yolo-SPM achieves the theoretical maximum possible value for the detection performance and with the CC-SPM model, a high value is obtained as well.

The length of the DP&OC-optimized flight trajectory for route reconnaissance with the CC-SPM performance model is about 5.5 km. In contrast, the trajectory resulting from the Yolo-SPM is approximately 3.3 km long. Both trajectories are shown in Figure 18. The long straight trajectory segments can be explained by the fact that the trajectory optimization is based on a discrete cartesian grid.

### 3.3. Area Reconnaissance Scenario

**Figure 19:**
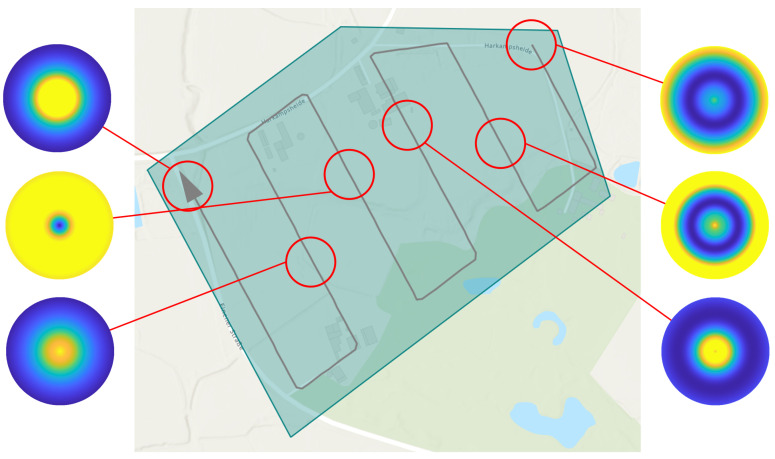
Illustration of the area reconnaissance scenario. The green line marks the sensor footprint path within the green reconnaissance area. Several perception maps resulting from the CC-SPM model illustrate the detection performance along the footprint path. The red lines indicate the positions of the perception maps along the sensor path. The color-coding of the different perception maps corresponds to the predicted detection performance.

In Figure 19, the area for vehicle detection is shown as a green colored zone. This area contains, similar to the route reconnaissance scenario, both rural and urban regions. The sensor footprint path was calculated in advance using coverage path planning. It is meander-shaped and has a length of approximately 3 km. The path is drawn as a green line within the reconnaissance area. Additionally, several perception maps resulting from the CC-SPM performance model are depicted.

#### 3.3.1. NMPC Trajectory Optimization

**Figure 20:**
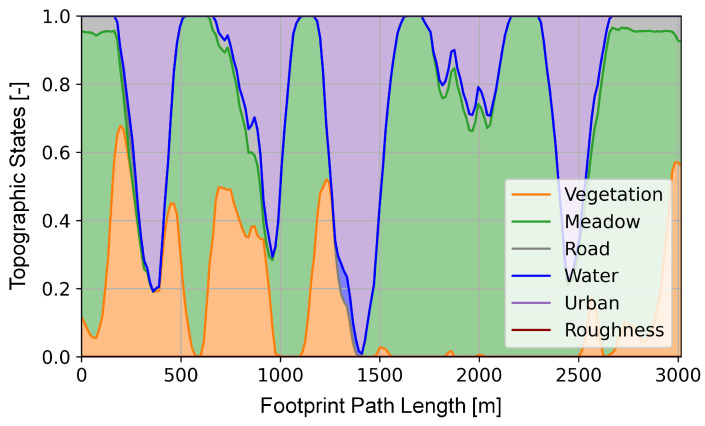
Topographic states resulting from the area reconnaissance scenario.

The atmospheric states for the CC-SPM sensor performance model are identical to the route reconnaissance settings listed in Table 9. The same applies to the atmospheric states for the Yolo-SPM, whose settings are summarized in Table 10. Figure 20 displays the topographic states along the sensor footprint path of the reconnaissance area. The large change in the topographic conditions along the footprint path is the cause of a high variation among the perception maps, which are depicted in Figure 19.

**Figure 21:**
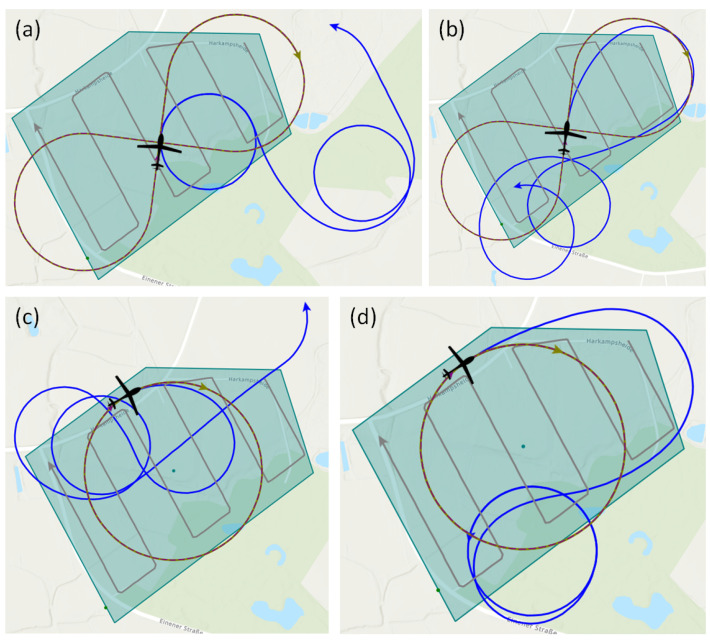
Trajectory optimization for the area reconnaissance scenario with sensor performance model CC-SPM in (**a**,**c**) and with Yolo-SPM in (**b**,**d**). The blue line indicates the NMPC-optimized reference trajectory and the light green line marks the benchmark trajectory. The starting points of both trajectories are identical and depicted by the aircraft symbol. In (**a**,**b**), the Figure-8 pattern is used, whereas in (**c**,**d**), the Circle benchmark pattern is applied.

The results of the trajectory optimization for the Figure-8 benchmark pattern are shown in (a) and (b), and the Circle pattern in (c) and (d) in Figure 21. In (a) and (c), the reference trajectories were optimized for the CC-SPM performance model, whereas in (b) and (d), they were optimized for the Yolo-SPM model.

Table 13 summarizes the simulation results for the area reconnaissance scenario with NMPC-optimized reference trajectory and the benchmark trajectory. The calculation of the *maximum average detection performance* and the *average detection performance* is carried out similarly to the description in Section 3.2.1. With the NMPC-optimized reference trajectory and the CC-SPM performance model, an average increase in detection performance of 3.71% can be achieved for area reconnaissance. With the Yolo-SPM performance model, an improvement of 4.86% is gained. Here, the length of the flight trajectory is about 3.5 km.

If we omit the separation into route and area reconnaissance, the average increase in detection performance by the NMPC-optimized reference trajectory compared to the benchmark trajectory is 4.09% with the CC-SPM performance model and 4.88% with the Yolo-SPM model. If all 24 simulation results are considered equally without differentiating between route and area reconnaissance or sensor performance models, the NMPC-optimized trajectory yields an increase in detection performance of 4.48% compared to the benchmark trajectory.

#### 3.3.2. DP&OC Trajectory Optimization

The results of the simulation with DP&OC are summarized in Table 14. Similar to the results in Table 12, it can be seen that the trajectory optimization with the Yolo-SPM achieves the theoretical maximum detection performance. A high value is also achieved using the CC-SPM performance model.

**Figure 22:**
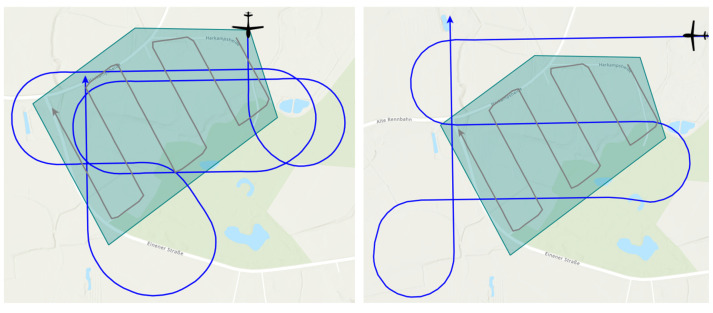
Illustration of the DP&OC-optimized trajectories for the area reconnaissance scenario with sensor performance models CC-SPM (**left**) and Yolo-SPM (**right**). The blue line depicts the UAV flight trajectory and the green line marks the sensor footprint on the ground.

In the case of area reconnaissance with the CC-SPM performance model, the length of the flight trajectory is approximately 6.3 km. In comparison, the optimized trajectory for the Yolo-SPM is about 5.3 km long. Both trajectories are depicted in Figure 22.

### 3.4. Computational Effort

The reference trajectories that are generated using sensor-model-based trajectory optimization are calculated in advance of the actual reconnaissance process and act as setpoint inputs. Therefore, the computation of these optimized trajectories is carried out prior to the execution of the flight and are therefore not subject to any real-time requirements. The optimization of the computation time was therefore not the focus of this work. Nevertheless, we would like to briefly mention the computational effort: The computations were performed on a desktop PC with a six-core processor running at 3.3 GHz. As mentioned in Section 2.7, the program code is implemented in C++ and Python. The computation time of the trajectories with NMPC optimization took about 30 s whereas the computation using DP&OC was about 15 min for route reconnaissance and about 20 min for area reconnaissance.

## 4. Discussion

By using our approach for sensor-model-based trajectory optimization, we were able to show that an increase in detection performance of approximately 4.5% on average was achieved with trajectory optimization using nonlinear model predictive control. With dynamic programming optimized reference trajectories, we even obtained detection performances that are equal or close to the theoretical maximum detection performance values.

Using the reference trajectories obtained by DP&OC optimization, a level of detection performance can be achieved that exceeds the average detection performance of both the benchmark and the NMPC-optimized trajectory. For the reference trajectories optimized for the Yolo-SPM model, even the theoretical maximum detection performance for route and area reconnaissance is achieved. However, this high detection performance comes at the cost of a significantly longer flight trajectory, which also increases the reconnaissance duration. For route reconnaissance, the flight duration increases by a factor of 1.3 to 2.1, depending on the sensor performance model used. For area reconnaissance, the flight duration increases by a factor of 1.5 to 1.8. Therefore, for mission planning, it must be weighed whether the gain in detection performance justifies the increase in reconnaissance duration.

The comparison of the computation time is intended to indicate that the generation of a global optimal reference trajectory with DP&OC optimization is associated with a computational effort about 40 times higher than for NMPC-optimized trajectories. It is therefore highly dependent on the application case, which optimization method shall or can be applied. We assume that the computation time for the DP&OC optimization could significantly be reduced by a high parallelization of the dynamic programming task. Alternatively, the resolution of the discretization can be reduced in order to vastly decrease the number of computations.

## 5. Conclusions

In this paper, we utilized sensor-model-based trajectory optimization to enhance detection performance in unmanned aerial reconnaissance. For this, we presented a newly developed sensor performance model, which maps relevant environmental states (including elevation angle and ground sample distance) to the expected detection performance for a perception chain with a YOLOv3 object detector. By utilizing the sensor performance model and optimization methods NMPC and DP&OC, we computed optimized reference trajectories for the UAV that are coordinated with the spatio-temporal positioning of the sensor footprint on the ground. By conducting several experiments in a simulation environment, with these reference trajectories, we achieved an increase in detection performance compared to the detection performance resulting from various benchmark trajectories. Furthermore, it could be shown for the DP&OC optimization method that the state transitions based on Dubins paths resulted in valid trajectories with consideration of curvature constraints.

In summary, we have verified that our approach of sensor-model-based trajectory optimization is capable of enhancing the resulting detection performance. Additionally, different requirements concerning perception (sensor, image processing algorithms), sensor platform (flight dynamics, flight envelope limitations), environment (daytime and season, illumination) and multiple mission aspects (reconnaissance area, high detection performance vs. reconnaissance duration) are considered for the optimization.

## Figures and Tables

**Table 1:** Parameter settings for coverage path planning.

Parameter	Setting	Remark
target ground sample distance $gsd_{ref}$	0.07 m	predefined
sensor resolution $R_{sens}$	1920 px	predefined
sweep width $w_{fp}$	134.4 m	from Equation (1)
footprint velocity $v_{fp}$	30 m/s	predefined
time step interval Δt	0.5 s	predefined
distance $d_{fp}$	15 m	from Equation (2)

**Table 2:** Atmospheric states comprising the aerial imagery dataset of [36].

Environmental State	Attributes
season	summer, autumn
daytime	day, night
visibility	clear, foggy
road condition	wet, dry
sky cover	covered, sunny

**Table 3:** Parameter settings for the generation of the perception maps.

Parameter	Setting	Remark
UAV altitude above ground $h_{agl}$	500 m	predefined
perception map diameter $d_{pm}$	2000 m	predefined

**Table 4:** Parameter settings for the path planning process.

Parameter	Setting	Remark
preview horizon time steps $M_{prev}$	25	predefined
time step interval Δt	0.5 s	from Table 1
uav setpoint velocity $v_{ref}$	35 m/s	predefined
path length $l_{path}$	437.5 m	from Equation (13)
number of paths *Z*	15	predefined

**Table 5:** Parameter settings for nonlinear model predictive control.

Parameter	Setting	Remark
prediction horizon *N*	10	predefined
maximum roll angle $\phi_{max}$	0.7 rad	from Equation (18)
setpoint roll angle $\phi^{ref}$	0 rad	predefined
maximum roll rate $\omega_{max}$	0.5 rad/s	from Equation (19)
setpoint roll rate $\omega^{ref}$	0 rad/s	predefined
minimum velocity $v_{min}$	33 m/s	from Equation (21)
maximum velocity $v_{max}$	37 m/s	from Equation (21)
setpoint velocity $v^{ref}$	35 m/s	predefined
maximum acceleration $a_{max}$	0.1 $m/s^{2}$	from Equation (20)
setpoint acceleration $a^{ref}$	0 $m/s^{2}$	predefined
diagonal weighting matrix *Q*	1, 1, 0.1, 0.1, 0.1	predefined
diagonal weighting matrix *R*	0.5, 0.5	predefined

**Table 6:** Parameter settings for the combined path planning and NMPC.

Parameter	Setting	Remark
weighting factor γ	0.8	predefined

**Table 7:** Parameter settings for Dubins path planning.

Parameter	Setting	Remark
UAV velocity *v*	35 m/s	predefined
maximum roll angle $\phi_{max}$	0.694 rad	predefined
gravitational acceleration *g*	9.81 $m/s^{2}$	
minimum turn radius $r_{min}$	150 m	from Equation (35)

**Table 8:** Parameter settings for dynamic programming and optimal control.

Parameter	Setting	Remark
UAV velocity *v*	35 m/s	from Table 7
minimum turn radius $r_{min}$	150 m	from Table 7
time step interval Δt	0.5 s	from Table 1
minimum Dubins path length $s_{min}$	17.5 m	from Equation (44)
maximum Dubins path length $s_{max}$	942.5 m	from Equation (45)
weighting factor $p_{w}$	0.5	predefined
number of yaw angles $m_{\psi ,n}$	12	predefined

**Table 9:** Parameter settings of the atmospheric conditions for the CC-SPM sensor performance model.

Parameter	Setting	Remark
time of day	16 h	predefined
month	June	predefined
cloud cover	25%	predefined
fog density	0%	predefined
precipitation	0%	predefined

**Table 10:** Parameter settings of the atmospheric conditions for the Yolo-SPM sensor performance model.

Parameter	Setting	Remark
daytime	day	predefined
season	autumn	predefined
visibility	clear	predefined
road condition	wet	predefined
sky cover	covered	predefined

**Table 11:** Predicted detection performance results for route reconnaissance with NMPC optimization.

	CC-SPM		Yolo-SPM	
	NMPC	Benchm.	NMPC	Benchm.
maximum average detection performance	0.972	0.972	0.936	0.936
average detection performance (abs.)	0.815	0.772	0.924	0.878
average detection performance (rel.)	83.88%	79.42%	98.66%	93.77%

**Table 12:** Predicted detection performance results for route reconnaissance with DP&OC optimization.

	CC-SPM	Yolo-SPM
maximum average detection performance	0.972	0.936
average detection performance (abs.)	0.910	0.936
average detection performance (rel.)	93.62%	100.00%

**Table 13:** Predicted detection performance results for area reconnaissance with NMPC optimization.

	CC-SPM		Yolo-SPM	
	NMPC	Benchm.	NMPC	Benchm.
maximum average detection performance	0.948	0.948	0.936	0.936
average detection performance (abs.)	0.846	0.810	0.933	0.887
average detection performance (rel.)	89.19%	85.48%	99.66%	94.80%

**Table 14:** Predicted detection performance results for area reconnaissance with DP&OC optimization.

	CC-SPM	Yolo-SPM
maximum average detection performance	0.948	0.936
average detection performance (abs.)	0.909	0.936
average detection performance (rel.)	95.89%	100.00%

## Data Availability

The data are not publicly available due to legal restrictions.

## Abbreviations

The following abbreviations are used in this manuscript:

AP	Average precision
BLOB	Binary large object
CPP	Coverage path planning
CC	Classification cascade
CC-SPM	Sensor performance model based on classification cascade object classifier
DPM	Deformable part model
DP&OC	Dynamic programming and optimal control
FN	False negative
FP	False positive
GIS	Geographic information service
GSD	Ground sample distance
IoU	Intersection over union
NMPC	Nonlinear model predictive control
OCP	Optimal control problem
PM	Perception map
TM	Template matching
TP	True positive
UAV	Unmanned aerial vehicle
Yolo-SPM	Sensor performance model based on YOLOv3 object detector

## References

[1] Zhang J., Huang H. (2021). Occlusion-Aware UAV Path Planning for Reconnaissance and Surveillance. Drones.

[2] Cambone S.A., Krieg K., Pace P., Linton W. (2005). Unmanned aircraft systems roadmap 2005–2030. Off. Secr. Def..

[3] Avola D., Foresti G.L., Martinel N., Micheloni C., Pannone D., Piciarelli C. (2017). Aerial video surveillance system for small-scale UAV environment monitoring. Proceedings of the 2017 14th IEEE International Conference on Advanced Video and Signal Based Surveillance (AVSS).

[4] Manfreda S., McCabe M., Miller P., Lucas R., Pajuelo Madrigal V., Mallinis G., Ben Dor E., Helman D., Estes L., Ciraolo G. (2018). On the Use of Unmanned Aerial Systems for Environmental Monitoring. Remote Sens..

[5] Langhammer J., Vacková T. (2018). Detection and Mapping of the Geomorphic Effects of Flooding Using UAV Photogrammetry. Pure Appl. Geophys..

[6] Nex F., Remondino F. (2014). UAV for 3D mapping applications: A review. Appl. Geomat..

[7] Feraru V.A., Andersen R.E., Boukas E. Towards an Autonomous UAV-based System to Assist Search and Rescue Operations in Man Overboard Incidents. Proceedings of the 2020 IEEE International Symposium on Safety, Security, and Rescue Robotics (SSRR).

[8] Qingqing L., Taipalmaa J., Queralta J.P., Gia T.N., Gabbouj M., Tenhunen H., Raitoharju J., Westerlund T. Towards Active Vision with UAVs in Marine Search and Rescue: Analyzing Human Detection at Variable Altitudes. Proceedings of the 2020 IEEE International Symposium on Safety, Security, and Rescue Robotics (SSRR).

[9] Erdos D., Erdos A., Watkins S.E. (2013). An experimental UAV system for search and rescue challenge. IEEE Aerosp. Electron. Syst. Mag..

[10] Sambolek S., Ivasic-Kos M. (2021). Automatic Person Detection in Search and Rescue Operations Using Deep CNN Detectors. IEEE Access.

[11] Jung H.K., Choi G.S. (2022). Improved YOLOv5: Efficient Object Detection Using Drone Images under Various Conditions. Appl. Sci..

[12] Howard R., Barrett S., Kunze L. (2021). Don’t Blindly Trust Your CNN: Towards Competency-Aware Object Detection by Evaluating Novelty in Open-Ended Environments. Proceedings of the 2021 IEEE International Conference on Robotics and Automation.

[13] Zwick M., Gerdts M., Stütz P. Sensor Model-Based Trajectory Optimization for UAVs Using Nonlinear Model Predictive Control. Proceedings of the AIAA SCITECH 2022 Forum; American Institute of Aeronautics and Astronautics.

[14] Zwick M., Gerdts M., Stutz P. Enhancing Detection Performance through Sensor Model-based Trajectory Optimization for UAVs. Proceedings of the 2021 IEEE/AIAA 40th Digital Avionics Systems Conference (DASC).

[15] Ru P., Subbarao K. (2017). Nonlinear Model Predictive Control for Unmanned Aerial Vehicles. Aerospace.

[16] Kamel M., Stastny T., Alexis K., Siegwart R., Koubaa A. (2017). Model Predictive Control for Trajectory Tracking of Unmanned Aerial Vehicles Using Robot Operating System. Robot Operating System (ROS).

[17] Garcia G.A., Keshmiri S.S., Stastny T. (2015). Robust and Adaptive Nonlinear Model Predictive Controller for Unsteady and Highly Nonlinear Unmanned Aircraft. IEEE Trans. Control Syst. Technol..

[18] Zhang Y., Wang W., Huang P., Jiang Z. (2019). Monocular Vision-based Sense and Avoid of UAV Using Nonlinear Model Predictive Control. Robotica.

[19] Ahmed K., Bousson K., Coelho M.d.F. (2021). A Modified Dynamic Programming Approach for 4D Minimum Fuel and Emissions Trajectory Optimization. Aerospace.

[20] Quintero S.A.P., Papi F., Klein D.J., Chisci L., Hespanha J.P. Optimal UAV coordination for target tracking using dynamic programming. Proceedings of the 49th IEEE Conference on Decision and Control (CDC).

[21] Harada A., Miyazawa Y. (2013). Dynamic Programming Applications to Flight Trajectory Optimization. IFAC Proc. Vol..

[22] MOKRANE A., BRAHAM A.C., CHERKI B., Recioui A. (2020). UAV Path Planning Based on Dynamic Programming Algorithm On Photogrammetric DEMs. Proceedings of the 2020 International Conference on Electrical Engineering (ICEE).

[23] Goerzen C., Kong Z., Mettler B. (2010). A Survey of Motion Planning Algorithms from the Perspective of Autonomous UAV Guidance. J. Intell. Robot. Syst..

[24] Betts J.T. (1998). Survey of Numerical Methods for Trajectory Optimization. J. Guid. Control Dyn..

[25] Hellert C., Koch S., Stutz P. Using Algorithm Selection for Adaptive Vehicle Perception Aboard UAV. Proceedings of the 2019 16th IEEE International Conference on Advanced Video and Signal Based Surveillance (AVSS).

[26] Acatay O., Sommer L., Schumann A., Beyerer J. (2018). Comprehensive Evaluation of Deep Learning based Detection Methods for Vehicle Detection in Aerial Imagery. Proceedings of the 2018 15th IEEE International Conference on Advanced Video and Signal Based Surveillance (AVSS).

[27] Andriluka M., Schnitzspan P., Meyer J., Kohlbrecher S., Petersen K., von Stryk O., Roth S., Schiele B. (2010). Vision based victim detection from unmanned aerial vehicles. Proceedings of the IEEE/RSJ International Conference on Intelligent Robots and Systems (IROS).

[28] Liu Y., Han K., Rasdorf W. (2022). Assessment and Prediction of Impact of Flight Configuration Factors on UAS-Based Photogrammetric Survey Accuracy. Remote Sens..

[29] Russ M., Stütz P. Airborne sensor and perception management: A conceptual approach for surveillance UAS. Proceedings of the 2012 15th International Conference on Information Fusion.

[30] Sandino J., Vanegas F., Gonzalez F., Maire F. (2020). Autonomous UAV Navigation for Active Perception of Targets in Uncertain and Cluttered Environments. Proceedings of the 2020 IEEE Aerospace Conference.

[31] Stecz W., Gromada K. (2020). Determining UAV Flight Trajectory for Target Recognition Using EO/IR and SAR. Sensors.

[32] Erickson L., LaValle S. A Simple, but NP-Hard, Motion Planning Problem. Proceedings of the AAAI Conference on Artificial Intelligence.

[33] Chandler P.R., Pachter M. Research issues in autonomous control of tactical UAVs. Proceedings of the 1998 American Control Conference. ACC (IEEE Cat. No.98CH36207).

[34] Choset H., Pignon P., Zelinsky A. (1998). Coverage Path Planning: The Boustrophedon Cellular Decomposition. Field and Service Robotics.

[35] Choset H. (2001). Coverage for robotics—A survey of recent results. Ann. Math. Artif. Intell..

[36] Krump M., Stütz P., Mazal J., Fagiolini A., Vasik P., Turi M. (2021). UAV Based Vehicle Detection with Synthetic Training: Identification of Performance Factors Using Image Descriptors and Machine Learning. Modelling and Simulation for Autonomous Systems.

[37] Jiao L., Zhang F., Liu F., Yang S., Li L., Feng Z., Qu R. (2019). A Survey of Deep Learning-Based Object Detection. IEEE Access.

[38] Zhao Z.Q., Zheng P., Xu S.T., Wu X. (2019). Object Detection With Deep Learning: A Review. IEEE Trans. Neural Netw. Learn. Syst..

[39] Du D., Qi Y., Yu H., Yang Y., Duan K., Li G., Zhang W., Huang Q., Tian Q. The unmanned aerial vehicle benchmark: Object detection and tracking. Proceedings of the European Conference on Computer Vision (ECCV).

[40] Redmon J., Farhadi A. (2018). Yolov3: An incremental improvement. arXiv.

[41] Krump M., Ruß M., Stütz P., Mazal J., Fagiolini A., Vasik P. (2020). Deep Learning Algorithms for Vehicle Detection on UAV Platforms: First Investigations on the Effects of Synthetic Training. Modelling and Simulation for Autonomous Systems.

[42] Zwick M., Koch S., Stütz P. Enhancing Detection and Tracking Performance Using Sensor-specific Flight Trajectory Generation for UAVs: A Conceptual Approach. Proceedings of the AIAA Scitech 2020 Forum.

[43] Bochkovskiy A., Wang C.Y., Liao H.Y.M. (2020). YOLOv4: Optimal Speed and Accuracy of Object Detection. arXiv.

[44] Nepal U., Eslamiat H. (2022). Comparing YOLOv3, YOLOv4 and YOLOv5 for Autonomous Landing Spot Detection in Faulty UAVs. Sensors.

[45] Hellert C. (2019). Algorithmenauswahl für den Adaptiven Sensoreinsatz an Bord Unbemannter Luftfahrzeuge. Ph.D. Thesis.

[46] Liu Y., Zhao Y. A virtual-waypoint based artificial potential field method for UAV path planning. Proceedings of the 2016 IEEE Chinese Guidance, Navigation and Control Conference (CGNCC).

[47] Allgöwer F., Badgwell T.A., Qin J.S., Rawlings J.B., Wright S.J., Frank P.M. (1999). Nonlinear Predictive Control and Moving Horizon Estimation—An Introductory Overview. Advances in Control.

[48] Mayne D. (2000). Nonlinear Model Predictive Control: Challenges and Opportunities. Nonlinear Model Predictive Control.

[49] Grüne L., Pannek J. (2017). Nonlinear Model Predictive Control.

[50] Kraft D. (1985). On Converting Optimal Control Problems. Computational Mathematical Programming.

[51] Bock H.G., Plitt K.J. (1984). A Multiple Shooting Algorithm for Direct Solution of Optimal Control Problems. IFAC Proc. Vol..

[52] Gerdts M. (2003). Direct Shooting Method for the Numerical Solution of Higher-Index DAE Optimal Control Problems. J. Optim. Theory Appl..

[53] Schittkowski K., Yuan Y.X., Cochran J.J. (2011). Sequential Quadratic Programming Methods. Wiley Encyclopedia of Operations Research and Management Science.

[54] de Nicolao G., Magni L., Scattolini R. (2000). Stability and Robustness of Nonlinear Receding Horizon Control. Nonlinear Model Predictive Control.

[55] Mayne D.Q., Rawlings J.B., Rao C.V., Scokaert P. (2000). Constrained model predictive control: Stability and optimality. Automatica.

[56] Allgöwer F., Findeisen R., Nagy Z.K. (2004). Nonlinear model predictive control: From theory to application. J. Chin. Inst. Chem. Eng..

[57] Findeisen R., Allgöwer F. An introduction to nonlinear model predictive control. Proceedings of the 21st Benelux Meeting on Systems and Control, 2002.

[58] Dubins L.E. (1957). On Curves of Minimal Length with a Constraint on Average Curvature, and with Prescribed Initial and Terminal Positions and Tangents. Am. J. Math..

[59] Reeds J., Shepp L. (1990). Optimal paths for a car that goes both forwards and backwards. Pac. J. Math..

[60] Chitsaz H., LaValle S.M. Time-optimal paths for a Dubins airplane. Proceedings of the 2007 46th IEEE Conference on Decision and Control.

[61] Lugo-Cardenas I., Flores G., Salazar S., Lozano R. Dubins path generation for a fixed wing UAV. Proceedings of the 2014 International Conference on Unmanned Aircraft Systems (ICUAS).

[62] Owen M., Beard R.W., McLain T.W., Valavanis K.P., Vachtsevanos G.J. (2015). Implementing Dubins Airplane Paths on Fixed-Wing UAVs. Handbook of Unmanned Aerial Vehicles.

[63] LaValle S.M. (2006). Planning Algorithms.

[64] Shkel A.M., Lumelsky V. (2001). Classification of the Dubins set. Robot. Auton. Syst..

[65] Boissonnat J.D., Cerezo A., Leblond J. Shortest paths of bounded curvature in the plane. Proceedings of the Proceedings 1992 IEEE International Conference on Robotics and Automation.

[66] Bellman R. (1972). Dynamic Programming.

[67] Bertsekas D.P. (2017). Dynamic Programming and Optimal Control.

[68] Quigley M., Conley K., Gerkey B., Faust J., Foote T., Leibs J., Berger E., Wheeler R., Ng A.Y. ROS: An open-source Robot Operating System. Proceedings of the ICRA Workshop on Open Source Software, 2009.

